# Demographic-Driven Wastewater-Based Epidemiology: Refining Population Estimates for Enhanced Chemical Monitoring

**DOI:** 10.1021/acs.est.5c08178

**Published:** 2025-11-18

**Authors:** Kishore Kumar Jagadeesan, Megan Robertson, John Bagnall, Barbara Kasprzyk-Hordern

**Affiliations:** † Department of Chemistry, 1555 University of Bath, Claverton Down, Bath BA2 7AY, U.K.; ‡ 329237 Centre of Excellence in Water-Based Early Warning Systems for Health Protection, Bath BA2 7AY, U.K.; § Wessex Water, Bath BA2 7WW, U.K.

**Keywords:** wastewater-based epidemiology, population estimation, Bayesian modeling, NHS registration, chemical biomarkers, environmental risk

## Abstract

Wastewater-based epidemiology (WBE) is a vital tool for public health and environmental monitoring, yet accurate population estimation and demographic profiles within wastewater treatment plant (WWTP) catchments remain a challenge. This study integrates high-resolution 2021 UK Census data, NHS patient registration records, and WWTP chemical parameters to refine population estimates and assess demographic influences on wastewater composition. We compared four statistical models: Simple Approach (SA), Density-Adjusted Bootstrapping (BtD), Density-Adjusted Bootstrapping with Overlap (BtDO), and Bayesian Hierarchical Modeling (ByD), across four diverse UK catchments (12,000–960,000). The BtDO method provided the most consistent estimates, with relative uncertainties as low as 1.5% in larger catchments. Demographic analysis revealed that older populations and higher deprivations correlated with elevated pharmaceutical load (e.g., analgesics, *r* = 0.92, *p* < 0.01). Traditional load-based indicators (Chemical Oxygen Demand (COD) and Biological Oxygen Demand (BOD)) significantly overestimated population due to nonhuman contributions, particularly in agricultural areas. This framework, transferable to regions with spatially referenced data, enhances WBE precision for public health surveillance and environmental risk assessment.

## Graphical abstract

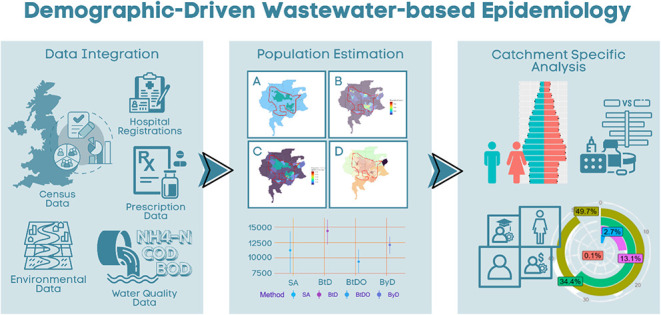

## 1 Introduction

Wastewater-based epidemiology (WBE) has emerged as a crucial tool for public health surveillance and environmental monitoring,^1,2^ enabling real-time,^3^ noninvasive tracking of community-wide behaviors,^4–6^ chemical exposures,^7,8^ and disease prevalence.^9–11^ However, its accuracy depends on reliable population size and demographic profile within wastewater treatment plant (WWTP) catchments.^12–15^ Two key metrics are used in WBE for the population size: population headcounts (*P*) and population equivalents (PE). Population estimate or headcounts (*P*), derived from census or administrative data, represent the actual number of individuals contributing to wastewater in a WWTP catchment.^15^ Accurate headcounts are essential for normalizing per-capita chemical loads, such as pharmaceuticals, to infer public health trends and behaviors. Errors in *P* can lead to misinterpretations of consumption patterns, undermining WBE’s reliability for surveillance. In contrast, Population Equivalents (PE) is a load-based metric reflecting the total organic load (i.e., 60 g per person per day of Biochemical Oxygen Demand, abbreviated as BOD) from residential, commercial, and industrial sources. PE is not a demographic headcount but an operational measure used by WWTPs for design, regulatory compliance, and infrastructure planning to account for all organic contributions, including nonhuman sources like agricultural runoff. Accurate PE values ensure that WWTPs are neither underloaded nor overloaded; underestimation can lead to overloaded systems and effluent permit violations, while overestimation may reduce process efficiency by underloading biological stages^14,16,17^ and ensuring compliance with environmental standards.^18^ Distinguishing *P* from PE is vital to avoid conflating demographic and load-based metrics, ensuring accurate WBE applications.

Accurate population estimation is challenging due to mismatches between WWTP catchments and census geographies,^19^ transient populations (e.g., commuters, tourists),^15^ and nonhuman contributions to wastewater (e.g., agricultural runoff). Water quality parameters like Biochemical Oxygen Demand (BOD),^20,21^ Chemical Oxygen Demand (COD), and ammonium (NH_4_–N) have been proposed as population proxies but are confounded by nonhuman sources.^15,22^ WBE has been developed over more than a decade of research by our group and others, advancing biomarker applications,^23–27^ modeling approaches,^28–30^ and translation into public and environmental health surveillance.^2,10,12,31–34^ Recent studies have advanced WBE by linking wastewater biomarkers to demographic trends^35–39^ For instance, O’Brien et al. (2020) in Australia used wastewater biomarkers to predict 37 socioeconomic characteristics,^37^ revealing higher pharmaceutical loads (e.g., tramadol) in deprived areas and age-related patterns (e.g., atenolol in older populations).^38–40^ Similar findings in Europe^41^ and Australia^42^ highlight WBE’s potential, yet few studies systematically compare advanced statistical models to quantify uncertainty in population estimates.^43^ Building on this foundation, our group recently demonstrated the use of multibiomarker approaches for population estimation, highlighting both the promise and limitations of biomarker-based approaches.^36^

The present study extends this work by evaluating statistical models that integrate high-resolution 2021 UK Census data, NHS patient registration records, and WWTP chemical data to refine catchment-level population size and demographic profiles. We compare four models: Simple Approach (SA), Density-Adjusted Bootstrapping (BtD), Density-Adjusted Bootstrapping with Overlap (BtDO), and Bayesian Hierarchical Modeling (ByD), across four UK catchments. Additionally, we evaluate biases in BOD, COD, and NH_4_–N as population proxies, and benchmark census-derived headcounts (*P*_Census_) against WWTP-reported residential (PE_WWTP,RP_), and total load-based (PE_WWTP,TP_) equivalents. Our objectives are as follows:

1. Refine population estimates using integrated 2021 UK census data and NHS patient registration records.
2. Assess demographic influences (age, deprivation) on wastewater chemical profiles.
3. Explore spatial variability in population movement by comparing *de jure* (census) and *de facto* (NHS GP registration) data.
4. Compare water quality-derived estimates against WWTP-reported benchmarks.

This framework, which is scalable to regions with spatially referenced data, supports advanced WBE applications for public health and environmental monitoring globally.

## 2 Methods

### 2.1 Study Sites

Four wastewater treatment plant (WWTP) catchments in Southwest England (WWTP A-D) were selected to represent contrasting urban and semiurban settings. Catchment population ranges from ∼12,000 to ∼960,000 and cover areas ∼15–2,000 km^2^. The largest catchment corresponds to the city of Bristol (WWTP A), followed by Bath (WWTP B), with a population of approximately 130,000. The smallest catchment corresponds to two smaller towns, Paulton (WWTP C) and Radstock (WWTP D), each with <30,000 residents. Catchment boundaries and their intersections with census Output Areas (OAs) are shown in Figure 1.

**Figure 1:**
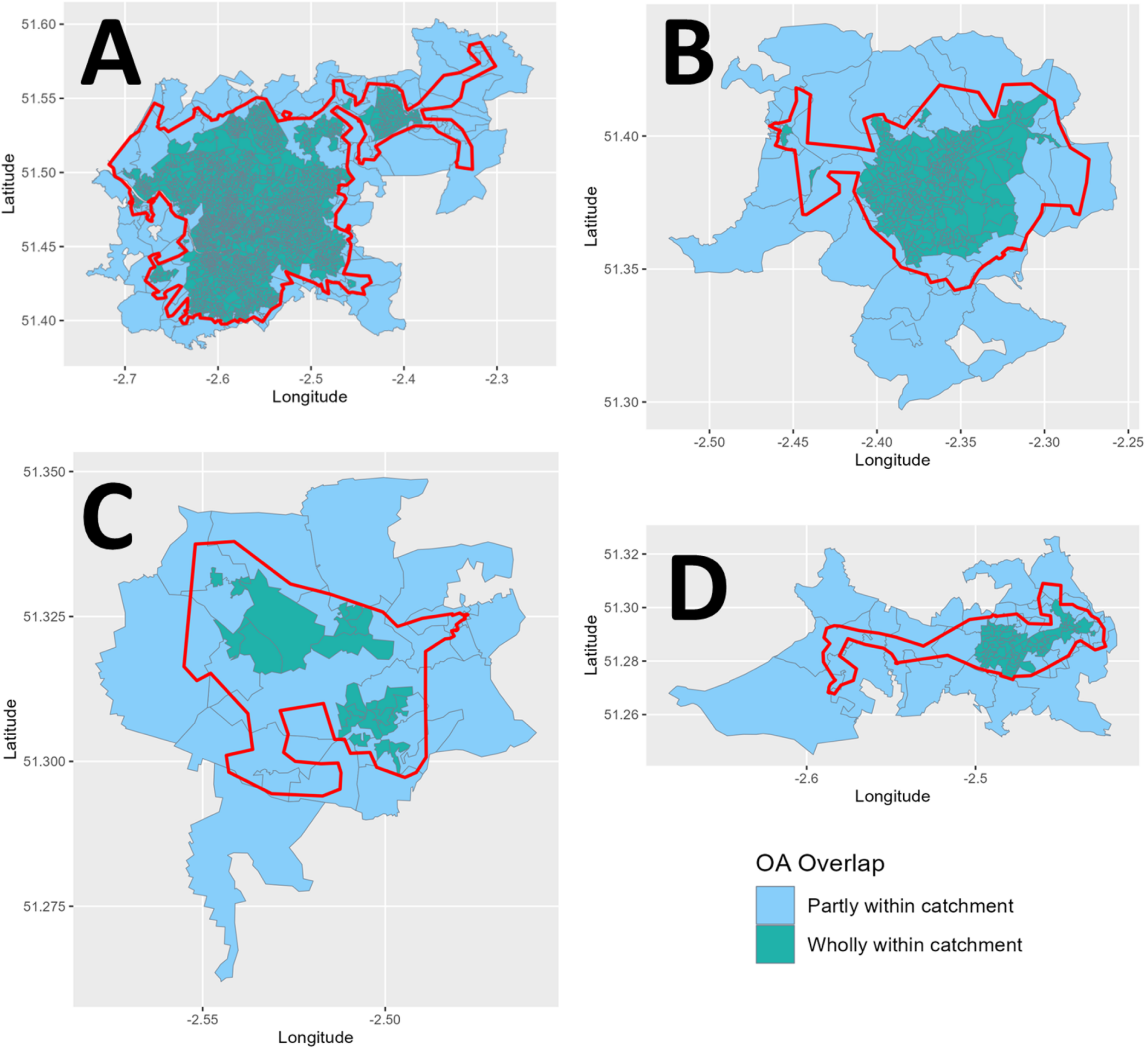
WWTP catchment boundary maps (red) overlaid onto the census map output area (OA) boundaries with OAs entirely (green) or partially (blue) within the WWTP catchment.

### 2.2 Data Sources and Integration

- 2021 UK Census Data: Population size was obtained from UK Census 2021 ^44^ at the Output Area (OA) level (∼100–625 individuals, 40–250 households). OA-level data include age, sex, and household indicators.
- NHS GP Registration Data: Population size was also obtained from NHS Digital quarterly General Practice (GP) registration data at Lower Layer Super Output Area (LSOA) level.^45^ The PrAna^30^ tool was used to locate GPs inside each WWTP catchment.
- WWTP Operational Data: Influent flow and chemical loads (NH4-N, BOD, COD) for the studied catchments were provided by the utility operator for the Census week (20–24 March 2021). Separately, we used utility-reported PE_WWTP_ values that are annualized and provided in two categories: residential (RP) and total (TP, includes trade effluent and tankered waste).
- Geospatial Data: WWTP catchment boundaries were supplied by the utility operator as GIS shapefiles. Census OAs and NHS LSOAs were spatially intersected with WWTP catchments using *R* to derive catchment-specific populations.

### 2.3 Terminology

Clear and consistent terminology was adopted to avoid ambiguity between headcount-based estimates and wastewater load-based equivalents, following recent recommendations in wastewater-based epidemiology (WBE) literature.^15,19^

- Population estimate (*P*): Refers exclusively to population size or headcount-based estimates derived from census data (*P*_Census_) or NHS GP registrations (*P*_NHS_).
- Population equivalent (PE): Refers exclusively to load-based equivalents, following engineering and utility conventions. These include load-based measures derived from influent wastewater parameters (PE_BOD_, PE_COD_, PE_Ammonia_) and utility-provided operational equivalents (PE_WWTP,TP_, PE_WWTP,RP_).

### 2.4 Population Estimation Approaches

Estimating the population served by a WWTP from administrative geographies is sensitive to boundary misalignment and aggregation effects. We therefore present a transparent, census-based baseline and two refinements that relax the uniformity assumption and quantify uncertainty (Figure 2). This design aligns with recent evaluations of population reporting in the WBE and with guidance on geospatial sensitivity.

**Figure 2:**
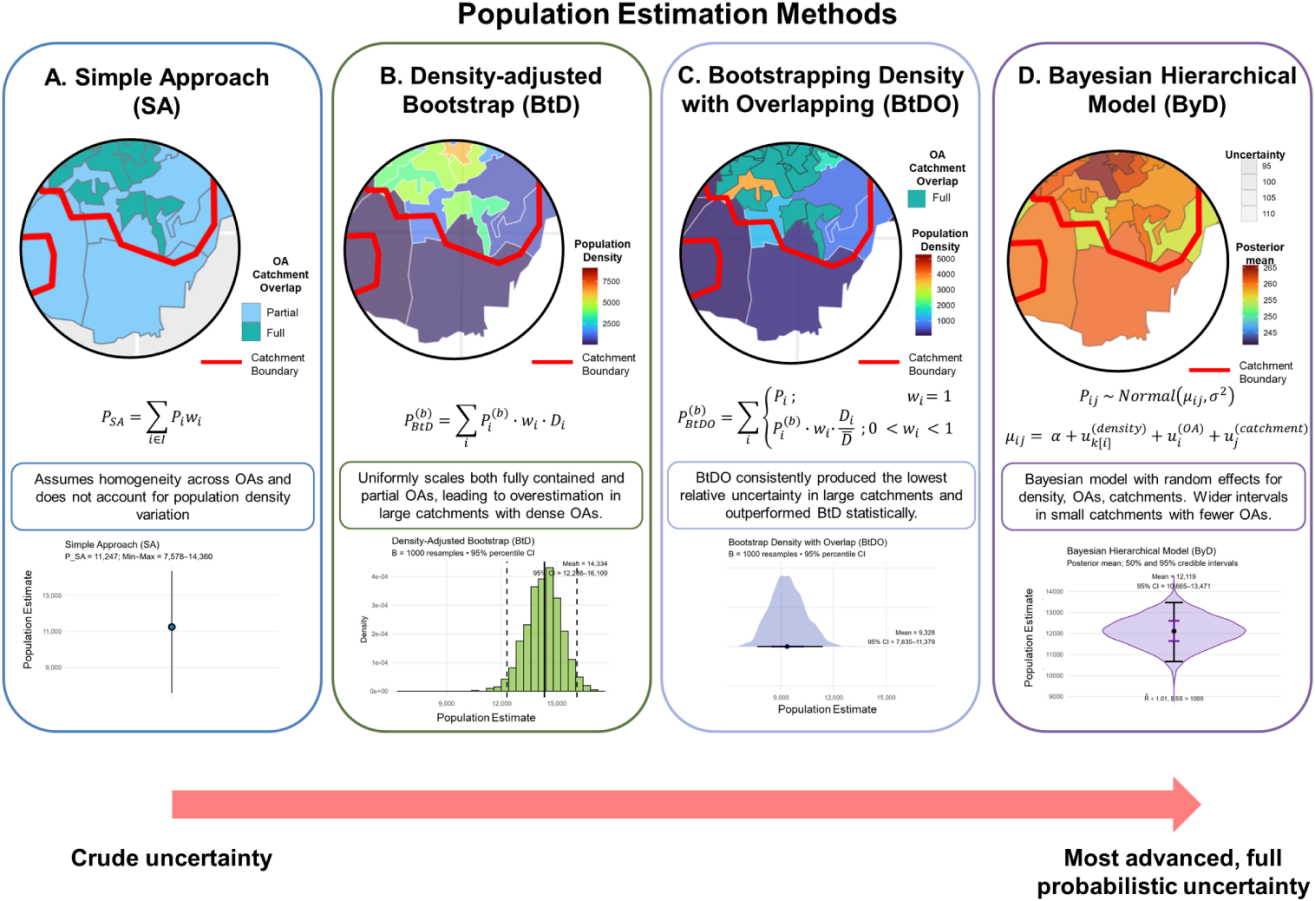
Conceptual overview of four population estimation methods (SA, BtD, BtDO, ByD) showing their assumptions, formulations, and representative outputs, highlighting trade-offs among simplicity, precision, and uncertainty quantification.

#### 2.4.1 Simple Approach (SA)

Figure 1 shows WWTP catchment boundaries (red) overlaid with Census Output Areas (OAs), distinguishing those that are fully contained (green) and partially intersecting (blue). Assuming uniform population distribution within each OA, the overlap-weighted census estimate is

$$P_{\mathrm{SA}}=\underset{i\in I}{\sum }P_{i}w_{i}$$

where *P*_*i*_ is the OA population, *I* is the set of all intersecting OAs, and *w_i_* ∈ [0,1] is the OA area fraction inside the catchment (1 if fully inside, 0 if outside). Bounding totals are

$$P_{min}=\sum_{i\in F}P_{i},P_{max}=\underset{i\in I}{\sum P_{i}}$$

with *F* the set of fully contained OAs. We report the spatial allocation uncertainty as

$$\mathrm{RU}\left(\%\right)=100\times \frac{P_{\max}-P_{\min}}{P_{\mathrm{SA}}}$$

#### 2.4.2 Density-Adjusted Approaches

##### 2.4.2.1 Density-Adjusted Bootstrap (BtD)

To improve the precision in catchments with partial OA overlap, we first implemented a density-adjusted bootstrap (BtD) method. For each OA *i* with population *P*_*i*_, area *A_i_*, and overlap fraction *w*_*i*_, density was defined as *D_i_* = *P_i_*/*A_i_*. OA contributions were scaled by both overlap and density:

$${\mathrm{contrib}}_{i}=P_{i}\cdot w_{i}\cdot D_{i}$$

Bootstrap resampling (*B* = 1000) was used to propagate uncertainty, and estimates were summarized as bootstrap means (*P*_BtD_), standard deviations, coefficients of variation (CV), and 95% percentile CIs.

##### 2.4.2.2 Density-Adjusted Bootstrapping with Overlapping (BtDO)

Because BtD may overweight fully contained high-density OAs, we refined the method to scale only partially overlapping OAs by their relative density (*D*_*i*_/*D̅*), *D̅* is the mean density across intersecting OAs in the bootstrap replicate. Fully contained OAs contributed unscaled.

$${\mathrm{contrib}}_{i}=\{\begin{array}{ll} P_{i} & w_{i}=1\\ P_{i}\cdot w_{i}\cdot \frac{D_{i}}{D̅} & 0<w_{i}<1 \end{array}$$

The total estimate for bootstrap iteration *b* was

$$P_{\mathrm{BtDO}}^{\left(b\right)}=\underset{i}{\sum }{{\mathrm{contrib}}_{i}}^{\left(b\right)}$$

And the overall estimate was

$$P_{\mathrm{BtDO}}=\frac{1}{B}\overset{B}{\underset{b=1}{\sum }}P_{\mathrm{BtDO}}^{\left(b\right)},,B=1000$$

Uncertainty was quantified by bootstrap SD, CV, and percentile 95% CI. By scaling only partial OAs, BtDO avoids double-counting density while ensuring that high-density partial areas contribute proportionally more than low-density areas. Further details and sensitivity checks are provided in Supporting Information Section S4.2.

#### 2.4.3 Bayesian Hierarchical Density Model (ByD)

To further improve the robustness of population estimates and capture variability beyond uniform or linear density assumptions, we applied a Bayesian hierarchical density model that treats population density, OAs, and WWTPs as random effects. This approach allows density effects to vary across discrete categories, improving flexibility in heterogeneous urban–rural contexts and avoiding overconfidence in a single functional form.

The model was specified as

$$P_{ij}∼\mathrm{Normal}\left(\mu_{ij},\sigma^{2}\right)$$

$$\mu_{ij}=\alpha +u_{k[i]}^{\left(\mathrm{density}\right)}+u_{i}^{\left(\mathrm{OA}\right)}+u_{j}^{\left(\mathrm{catchment}\right)}$$

where

- *P_ij_* is the contribution of OA *i* to catchment *j*,
- α is the global intercept,
-
$u_{k[i]}^{\left(\mathrm{density}\right)}∼\mathrm{Normal}\left(0,\sigma_{\mathrm{density}}^{2}\right)$ is the random effect for density stratum *k* (e.g., low, medium, and high),
-
$u_{i}^{\left(\mathrm{OA}\right)}∼\mathrm{Normal}\left(0,\sigma_{\mathrm{OA}}^{2}\right)$ is the random effect for OA *i*,
-
$u_{j}^{\left(\mathrm{catchment}\right)}∼\mathrm{Normal}\left(0,\sigma_{\mathrm{catchment}}^{2}\right)$ is the random effect for WWTP catchment *j*.

Random effects were assumed to be normally distributed with mean zero and group-specific variance. The models were fitted in *R* (brms; 4 chains × 4000 iterations; adapt_delta = 0.95 (to reduce divergent transitions)) with weakly informative priors (Normal­[0,10] for intercepts; Normal­[0,10] for random-effect SD). Convergence was confirmed by *R̂*< 1.01 and effective sample sizes >1000.

Posterior samples were summarized as *P*_ByD_ (posterior mean), 95% credible intervals (CrIs), root-mean-square error (RMSE), and coverage probability for each WWTP catchment. Further details are provided in Supporting Information Section S4.3.

### 2.5 NHS GP Registration-Based Estimates (*P*_NHS_)

Population headcounts from GP registrations (*P*_NHS_) were derived by aggregating LSOA-level GP registration data for LSOAs overlapping with each WWTP catchment. GP surgery information, such as the postcode and the number of people registered, was obtained from NHS Digital (https://digital.nhs.uk/). The PrAna^30^ tool was used to identify the GP surgeries present in each WWTP catchment.

### 2.6 Population Equivalent Estimation Using Water Utility Data

Load-based population equivalents from WWTPs (PE) were calculated using NH_4_–N, BOD, and COD using

$$\mathrm{PE}=\frac{C\times F}{E}$$

*C* is the influent concentration (mg/L) of the conventional indexes (COD, BOD, NH_4_–N) of the sewage treatment plant; *F* is the daily flow (L/day); and *E* is the emissions per-capita emission factor (BOD = 60 g/day/person, COD = 120 g/day/person, NH_4_–N = 7.7–8.5 g/day/person). These were benchmarked against *P*_Census_, PE_WWTP,RP_, and PE_WWTP,TP_.

### 2.7 Determination of Population Age, Sex, Household Deprivation, and People Travel to Work Profiles

Demographic breakdown (age, sex, household deprivation) and work travel patterns were derived from Census 2021 data at the OA level. Overlaps with WWTP catchments were weighted using the BtDO methodology. This provided catchment-specific profiles to contextualize population variability.

### 2.8 Evaluation and Comparison

For all estimations, Absolute Uncertainty, Relative Uncertainty (%), and Margin of Error were derived from confidence intervals or credible intervals. ANOVA with Tukey’s HSD posthoc test assessed differences across methods and catchments, incorporating OA overlap as a covariate.

## 3 Results and Discussion

### 3.1 Performance of Population Estimation Methods

Population estimates based on UK 2021 Census (*P*_Census_) were used to evaluate four estimation methods across the four WWTP catchments (A–D): Simple Approach (SA), Bootstrap Density (BtD), Bootstrap Density with Overlap (BtDO), and Bayesian Hierarchical Density (ByD). Table 1 summarizes estimates, 95% confidence intervals (CIs) for SA, BtD, and BtDO, and 95% credible intervals for ByD, alongside relative uncertainty metrics. Method performance was strongly influenced by catchment size and the proportion of OAs fully overlapped with WWTP boundaries.

**Table 1:** Population Estimates Based on Census 2021 Output Area (OA) and WWTP Catchment Map Overlap, Based on Simple Approach (SA), Bootstrap Density (BtD), Bootstrap Density with Overlap (BtDO), and Bayesian Hierarchical Density (ByD) with 95% Confidence Intervals (CIs) for SA, BtD, and BtDO, and 95% Credible Intervals for ByD, alongside Relative Uncertainty Metrics

WWTP	OA^a^ with 100% Overlap (count)	OA^a^ with 100% Overlap (%)	Method^b^	Population Estimate (persons)	CI^c^ Lower (persons)	CI^c^ Upper (persons)	Absolute Uncertainty (persons)	Relative Uncertainty (%)
A	2114	95.1%	SA	720862	702792	737774	17491.00	2.43
			BtD	738935	708011	769816	30902.54	4.18
			BtDO	706279	695351	717094	10871.07	1.54
			ByD	720556	634351	806830	86239.62	11.97
B	328	90.6%	SA	104826	99231	110894	5831.50	5.56
			BtD	112055	102189	122165	9988.12	8.91
			BtDO	99838	94044	106860	6407.73	6.42
			ByD	104283	89734	118766	14516.08	13.92
C	20	42.6%	SA	11074	6941	15037	4048.00	36.55
			BtD	16093	10421	21108	5343.90	33.21
			BtDO	9591	7687	11537	1924.75	20.07
			ByD	12093	9836	14318	2240.89	18.53
D	56	65.1%	SA	23620	18692	28706	5007.00	21.20
			BtD	29762	23430	35147	5858.30	19.68
			BtDO	20703	18096	23319	2611.21	12.61
			ByD	23747	20001	27482	3740.67	15.75

a OA: Output area.

b SA: Simple approach, BtD: Bootstrap density, BtDO: Bootstrap density with overlap, ByD: Bayesian hierarchical density.

c 95% confidence intervals (CIs) for SA, BtD, and BtDO, 95% credible intervals for ByD.

The SA, which weights OA populations by proportional overlap, yielded the widest CIs and highest relative uncertainties, particularly in small or fragmented catchments. For WWTP C (42% OA overlap), the *P*_Census_ was 11,074 (CI: 6,941–15,037; 36.6% uncertainty); for WWTP A (95.1% OA overlap), the *P*_Census_ was 720,862 (CI: 702,792–737,774; 2.43% uncertainty). While computationally efficient, SA lacks robustness in smaller or spatially fragmented catchments.

The BtD method, incorporating population density weighting across all OAs, improved performance in larger catchments (WWTP A: 738,935; CI: 708,011–769,816; 4.2% relative uncertainty) but showed excessive uncertainty in smaller ones. WWTP C (16,093; CI: 10,421–21,108) showed 33.2% relative uncertainty, and WWTP D (29,762; CI: 23,430–35,147) showed 19.7% relative uncertainty. These results confirm BtD’s reliability in large, dense catchments but limitations in smaller, sparsely populated areas.

The BtDO method, a novel refinement that applies density adjustment only to partially overlapping OAs while treating fully contained OAs as complete contributors, consistently outperformed SA and BtD. This approach reduced bias and tightened confidence intervals. For WWTP A, the *P*_Census_ was 706,279 (CI: 695,351–717,094), with only 1.54% relative uncertainty. Although performance declined in smaller catchments, 20.1% in WWTP C and 12.6% in WWTP D: 12.6%, BtDO provided the most efficient and robust estimates overall. Posthoc analysis confirmed significant improvement relative to that of BtD (*p* = 0.0079). Figures 3 and S5show BtDO’s narrower demonstration and confirm BCa and percentile intervals were identical across catchments (Figure S1), validating the robustness of bootstrap uncertainty quantification. Bland–Altman analysis further demonstrated a negligible systematic bias between BtDO and WWTP-derived population equivalents (Figure S4).

**Figure 3:**
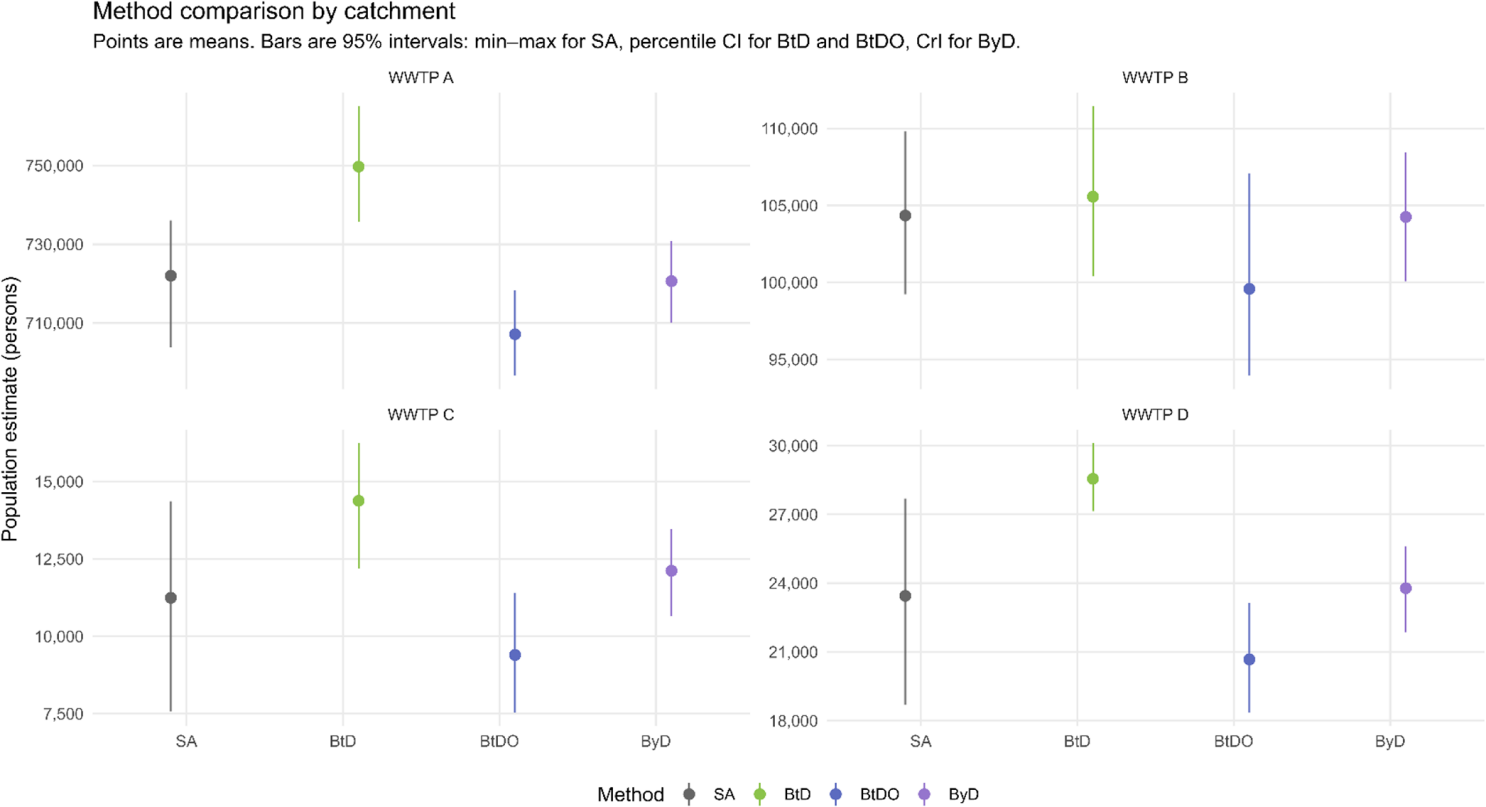
Population estimates across different methods for wastewater treatment plant (WWTP) catchments. The bar plot shows population estimates for each WWTP catchment area A–D using four different estimation methods: simple approach (SA), bootstrap density (BtD), bootstrap density with overlap (BtDO), and Bayesian density (ByD). Error bars represent the 95% confidence intervals (CI) for SA, BtD, and BtDO, and 95% credible intervals for ByD, illustrating the uncertainty associated with each estimate.

The ByD model, with random effects for density, OAs, and catchment-specific factors, offered posterior distributions with interpretable credible intervals, reflecting both data-driven variability and parameter uncertainty. For WWTP A, the estimate was 720,556 (credible intervals: 634,351–806,830; 12% relative uncertainty); for WWTP C, it was 12,093 (CI: 9,836–14,318; 18.5% relative uncertainty) reflecting sparse overlap. Prior predictive checks confirmed that weakly informative priors did not drive results, as census totals fell within prior distributions across all catchments (Figure S2B). Sensitivity analysis further indicated stability to alternative priors, with posterior means varying by <5% (Figure S2A). Although computationally more intensive, ByD captures hierarchical variation and offers a richer representation of uncertainty, making it particularly suited to settings where robust probabilistic inference is required (e.g., national surveillance and longitudinal monitoring).

Cross-method comparisons (Figures S3 and S5) highlight trade-offs. SA provides bounds but with high uncertainty. BtD improves density scaling but can inflate totals. BtDO balances precision and robustness, particularly in high-overlap catchments, while ByD offers richer uncertainty representation at a higher computational cost. Agreement of BtDO with WWTP treatment-plant equivalents (Figure S4) further strengthens confidence.

Overall, BtDO emerges as the most reliable and practical estimator for routine WBE population estimation, while ByD provides a complementary probabilistic framework suited for applications requiring inference under uncertainty. Spatial overlap remains a key determinant of precision, suggesting future work should focus on harmonizing catchment and census boundaries and leveraging ancillary data sets.^46–48^

### 3.2 Alignment with Census and GP Registration Populations

Comparison of census-derived estimates (*P*_Census_) and NHS GP registration data (*P*_NHS_) revealed discrepancies ranging from −0.3% to 31.7% (Table 3). Agreement was the strongest for WWTP A, where well-aligned OA and LSOA boundaries (95.1% and 87.2% overlap, respectively) produced only a 3% difference. In contrast, WWTP C showed 31.7% higher *P*_NHS_ than *P*_Census_, driven by low OA overlap (42.6%) and the complete absence of fully overlapping LSOAs. These findings are consistent with previous studies, showing that larger populations yield more stable estimates across methods, while smaller populations display greater variability.^15,19^

Impact of the Mobility Patterns: Population mobility further explained the discrepancies. WWTPs A and B exhibited higher proportion of residents working from home (37.3% and 43.5%, respectively; Figure S7), contributing to more stable daytime populations. By contrast, WWTPs C and D reported lower work-from-home rates (30.1% and 21.3%) and higher commuter proportions traveling 10–20 km (24.7% and 22.4% residents), introducing greater temporal variability. Notably, ∼17% of residents in WWTP C and D reported working outside the UK, complicating headcount estimation. While partially captured by GP registration data, transient and highly mobile populations remain under-represented in census counts, leading to the observed discrepancies. These results underscore the importance of integrating census and administrative data sets to capture commuting behaviors, transient populations, and spatial mismatches, factors that are especially critical in smaller and more mobile catchments.

### 3.3 Water Quality Indicators as Population Equivalents

Traditional load-based proxies (NH_4_–N, BOD, and COD) systematically overestimated the *P*_Census_ across the sites (Table 2, Figure S8). COD was the most biased, with overestimates up to +198% in agricultural catchments (WWTP C). BOD performance was inconsistent, underestimating by 11% in WWTP B but overestimating by 116% in WWTP C. NH_4_–N aligned most closely with the total operational PE (PE_WWTP,TP_), differing by less than ±6.6% across sites, confirming that ammonia primarily represents nitrogenous loading from residential and nonresidential contributors.

**Table 2:** Population Estimates for Four WWTP (A, B, C, and D) Derived from Ammonia (NH_4_–N), Biological Oxygen Demand (BOD), Chemical Oxygen Demand (COD) Measurements, and Population Estimate from Wastewater Treatment Plants (WWTPs)

WWTP	PE_Ammonia_ (±SD)	PE_BOD_ (±SD)	PE_COD_ (±SD)	PE_WWTP_ (RP)	PE_WWTP_ (TP)	*P*_Census_ ^a^ (±SD)
A	908373 ± 37581	886840 ± 76654	1220835 ± 186957	740086	966296	721658 ± 13378
B	128364 ± 1524	93740 ± 9660	129324 ± 14646	115850	125932	105250 ± 5057
C	12980 ± 623	26412 ± 10439	36452 ± 11498	12137	12300	12213 ± 2783
D	29860 ± 1540	25413 ± 3998	37055 ± 5069	27034	28021	24458 ± 3805

a Mean estimate from the population estimates based on different methods.

Land use analysis (Figure S9) confirmed that COD and BOD were strongly correlated with agricultural area (*r* = 0.77 and *r* = 0.76, respectively), highlighting nonhuman contributions. By contrast, NH_4_–N correlated closely with PE_WWTP,TP_ (*R* ^2^ ∼ 0.99). These findings reinforce that *P*_Census_ should be used to normalize per-capita pharmaceutical loads, while PE_WWTP,TP_ or PE_Ammonia_ are more appropriate for operational benchmarking of wastewater load.^15,49,50^

Land-use distributions varied markedly across the four WWTP catchments (Figure S9). WWTP A was characterized by high proportions of transport/utilities and industrial/commercial land, reflecting its urban and port-associated infrastructure. WWTP B showed a balanced mix, with substantial residential areas and gardens alongside transport networks, consistent with a dense urban setting. In contrast, WWTP C and WWTP D were dominated by agriculture (>50%), with smaller contributions from residential and recreational land. These differences illustrate the peri-urban gradient across sites: WWTP A and WWTP B represent dense, built-up catchments where wastewater inflows align closely with census-derived populations, while WWTP C and WWTP D highlight mixed rural settings where extensive agricultural land dilutes wastewater signals relative to the resident population.

Land use correlation analysis (Figure S10) confirmed that COD and BOD were strongly correlated with agricultural area (*r* = 0.77 and *r* = 0.76, respectively), highlighting nonhuman contributions. The strong correlation between BOD and COD (*r* = 0.95) confirmed their complementary roles as indicators of organic loading. By contrast, NH_4_–N correlated closely with PE_WWTP_(TP) (*R* ^2^ ∼ 0.99). These findings reinforce that *P*_Census_ should be used to normalize per-capita pharmaceutical loads, while PE_WWTP,TP_ or PE_Ammonia_ are more appropriate for operational benchmarking of wastewater load.^15,49,50^ While exploratory, given the small number of sites, the results highlight the importance of catchment land use in shaping wastewater-derived water quality signals.

### 3.4 Benchmarking against WWTP-Reported Populations

Benchmarking against WWTP-reported population equivalents was necessary to clarify whether census-based headcounts align with operational measures of population, which are often conflated in WBE applications. We compared *P*_Census_ with two operational benchmarks: the residential population equivalent (PE_WWTP,RP_) and the total operational equivalent (PE_WWTP,TP_).

*P*_Census_ vs PE_WWTP_,_RP_: Discrepancies were modest, ranging from −0.6% at WWTP C to +10.5% at WWTP B. This indicates that *P*_Census_ provides a reasonable proxy for resident contributors to domestic wastewater, although minor deviations reflect small-scale boundary mismatches or incomplete registration coverage.

*P*_Census_ vs PE_WWTP_,_TP_: Larger discrepancies were observed where nonresidential inputs were substantial. At WWTP A, *P*_Census_ underestimated PE_WWTP,TP_ by 244,638 people (−25.3%), attributable to 45,309 commercial, 142,190 tankered domestic, and 32,125 trade effluent inputs. This demonstrates that TP reflects total system loading, not a demographic headcount, and should be interpreted as an operational rather than a population measure.

PE_WWTP,TP_ vs water-quality equivalents: Among hydrochemical proxies, PE_Ammonia_ showed the strongest agreement with PE_WWTP,TP_, with discrepancies within ± 6.6% and *R* ^2^ ∼ 0.99. This consistency reflects ammonia’s stability in sewage and its dominant origin in urea metabolism,^51^ though contributions from nonresidential sources remain. By contrast, BOD- and COD-based equivalents exhibited higher variability, with systematic overestimation in agricultural catchments (e.g., COD at WWTP C). These results confirm ammonia as a reliable load-based proxy but highlight the need for correction factors in the interpretation of COD or BOD Table 3.

**Table 3:** Population Movement Estimates

WWTP	PE_NHS_ (±SD)	PE_Census_ (±SD)^a^ ^a^	% Difference	LSOA 100% Overlap (%)
A	718797 ± 1888	721658 ± 13378	3.02	87.2%
B	107976 ± 808	105250 ± 5057	–0.28	73%
C	14577 ± 93	12213 ± 2783	31.67	0%
D	25133 ± 96	24458 ± 3805	6.45	25%

a Mean estimate from the population estimates based on different methods.

### 3.5 Prescription Data as a Proxy for Demographic Dynamics

Integration of prescription data with census-based population estimates provided a dynamic perspective on demographic and socioeconomic influences shaping pharmaceutical consumption in wastewater catchments. Both “age structure” and “household deprivation” emerged as strong modifiers of drug usage, underscoring the need to contextualize WBE signals with local population characteristics.

#### 3.5.1 Age Group Distribution

Population profiles varied across the four catchments (Table S2, Figure 5). Prescription trends were strongly age-dependent. Beta-blocker use correlated positively with older populations (Figure 4) (65+ years: *r* = 0.83, *p* < 0.05), (50–64 years: *r* = 0.78, *p* < 0.05) and negatively with younger adults (20–49 years: *r* = −0.88, *p* < 0.005). Analgesic use followed a similar pattern, correlating with 50–64 years (*r* = 0.92, *p* < 0.01) and 65+ years (*r* = 0.74, *p* < 0.05), but negatively with 20–49 years (*r* = −0.89, *p* < 0.01). These age-dependent patterns translated into catchment-level differences: naproxen and gabapentin were elevated in catchments C and D, consistent with their older populations^2^ and used in treating arthritis and chronic pain (Figure S11). By contrast, paracetamol exhibited broad, nonspecific use across all age groups. These findings confirm that skewed age distributions can bias WBE-derived consumption estimates unless explicitly accounted for.

**Figure 4:**
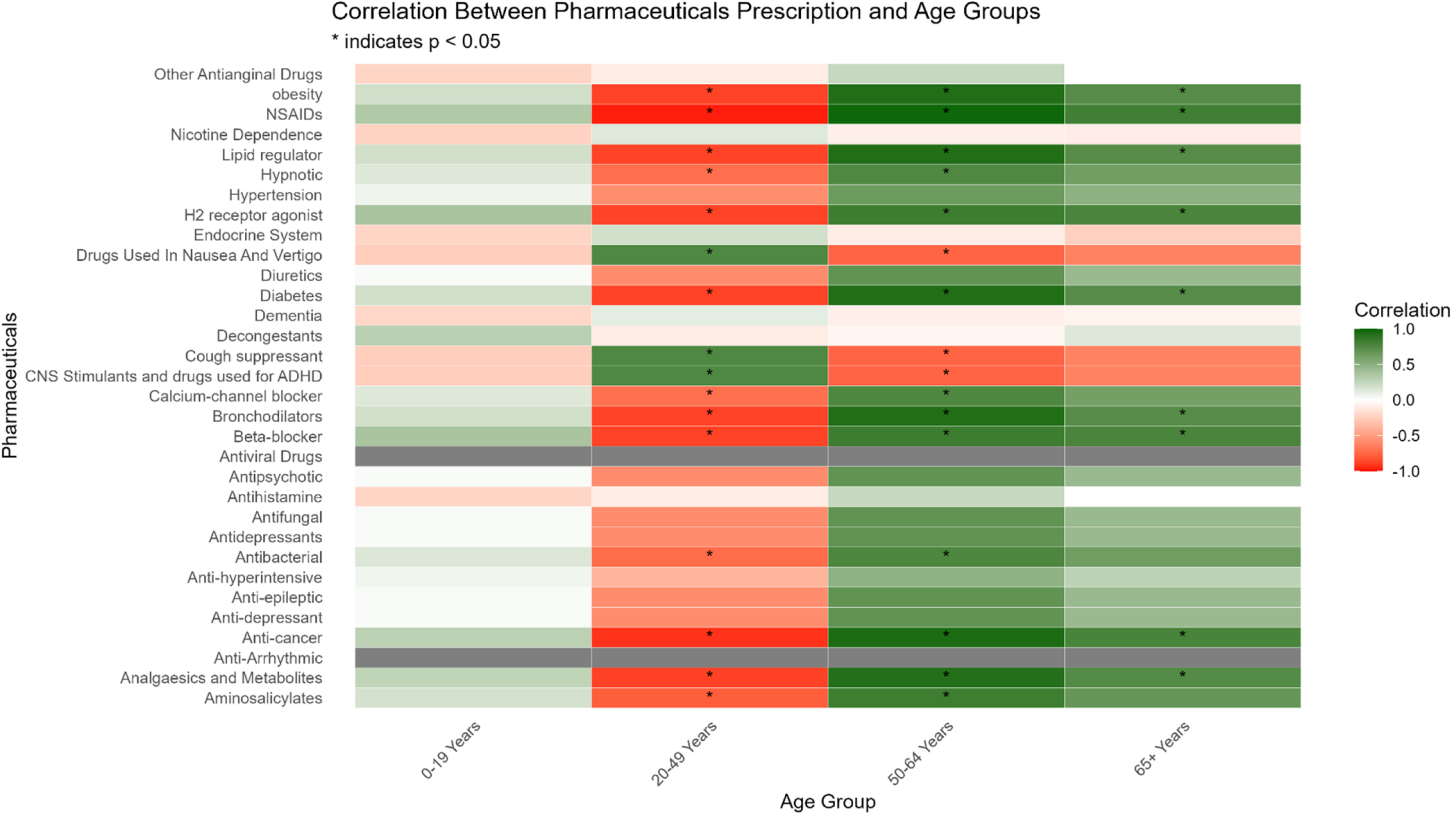
Correlation between pharmaceutical prescription and age groups across studied wastewater treatment plant (WWTP) catchments. The heatmap shows the correlation coefficients (*r*) between age groups (*x*-axis) and drug families (*y*-axis). Colors represent the strength and direction of correlations, with green indicating positive correlations, red indicating negative correlations, and white indicating no correlation. Significant correlations (*p* < 0.05) are denoted by asterisks (*).

#### 3.5.2 Household Deprivation

Socioeconomic disparities also influenced pharmaceutical consumption patterns (Table 4; Figure 5). WWTP B had the lowest deprivation, with 54.8% of households unaffected, while WWTP D was the most deprived (only 49.7% of households unaffected, and 34.4% with one-dimensional and 13.1% with two-dimensional deprivation). Severe deprivation (three or four dimensions) remained rare (<3% across sites). Prescription loads mirrored these patterns: citalopram (antidepressant) consumption was more than double in WWTP D (2.6 × 10^–4^ kg/inhabitant/year) compared to WWTP A (1.04 × 10^–4^ kg/inhabitant/year). Diazepam (hypnotic) and metformin (diabetes treatment) use was similarly elevated in WWTP D and C (Figure S11), consistent with higher burdens of mental health and chronic disease in deprived communities.

**Table 4:** Percentage of Households Experiencing Different Dimensions of Deprivation at Each Site Based on Data from the 2021 Census

WWTP	Four Dimension	Three Dimension	Two Dimension	One Dimension	No Dimension
A	0.2 ± 0	3.3 ± 0	13 ± 0.06	32.1 ± 0.06	51.4 ± 0.06
B	0.2 ± 0	2.4 ± 0	11.1 ± 0.1	31.5 ± 0	54.8 ± 0.15
C	0.1 ± 0	1.9 ± 0.2	11.2 ± 0.21	32.9 ± 0.64	53.9 ± 0.55
D	0.1 ± 0	2.7 ± 0.12	13.1 ± 0.4	34.4 ± 0.15	49.7 ± 0.35

**Figure 5:**
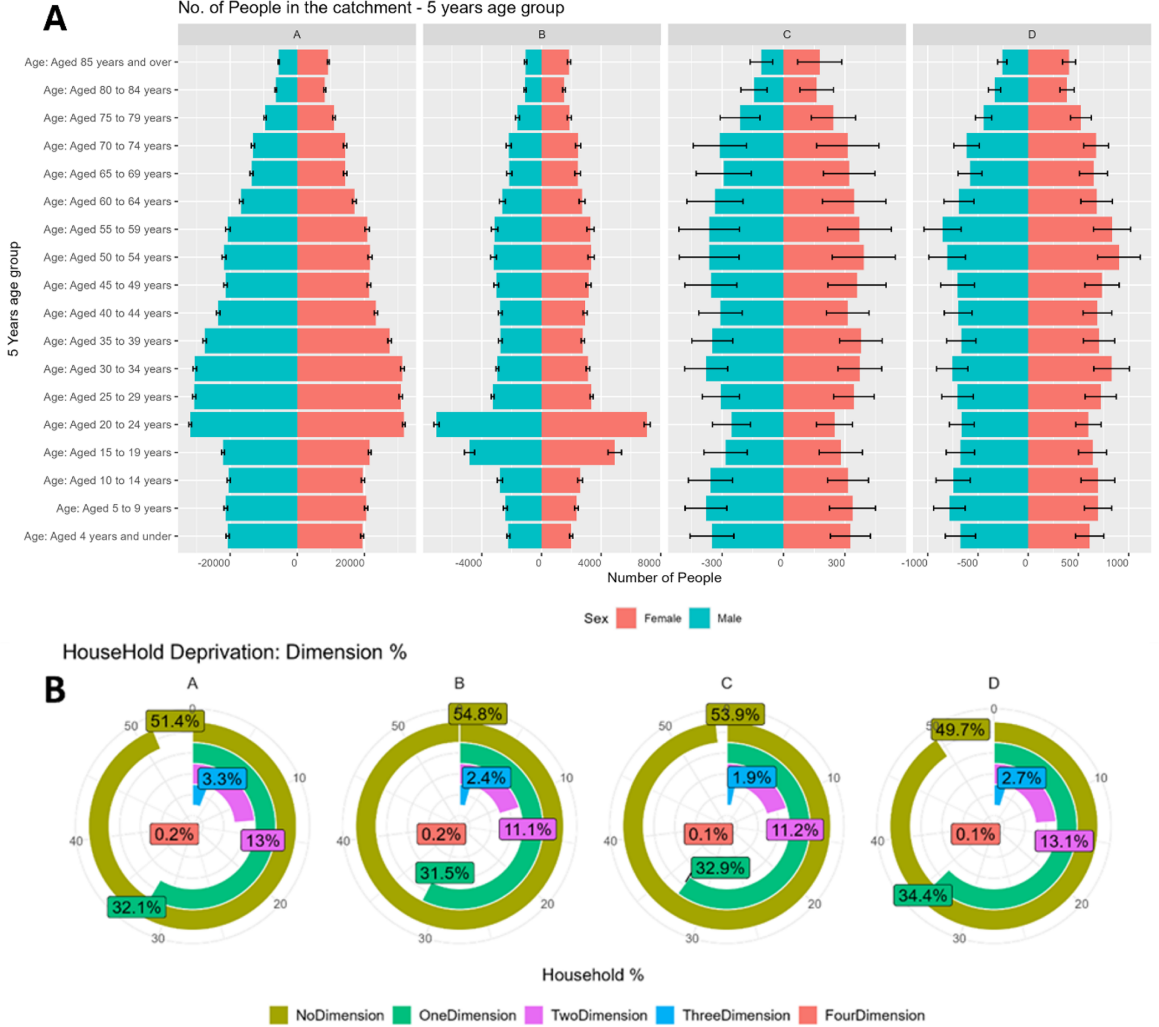
A. Five-year age group distribution by sex at each WWTP. B. Percentage of households experiencing different dimensions of deprivation at each site, based on data from the 2021 Census.

Together, these findings underscore that both age structure and deprivation strongly influence prescription trends, and therefore modify WBE interpretations.^15^ Without accounting for demographic and socioeconomic heterogeneity, the risk of misattributing observed pharmaceutical loads. Integrating prescription records and census-derived profiles into population normalization frameworks offers a path toward more robust and equitable WBE-based surveillance.

#### 3.5.3 Limitations and Future Perspectives

This study demonstrates the value of integrating WWTP catchment boundaries, census data, NHS registration records, and prescription data to generate refined, catchment-specific population estimates and demographic profiles for the WBE. Several limitations remain, alongside clear opportunities for scaling.

- Geographic scope: Analyses are limited to four Southwest England catchments, while the framework is transferable, a broader application will require testing across diverse geographies.
- Temporal resolution: Census data provided a robust baseline but did not capture short-term population fluctuations. NHS registrations improve timeliness but were quarterly. At the national scale, integration with real-time indicators (e.g., daily flow, mobility data, or GP deregistration) could enable rolling population estimates, supporting weekly or monthly WBE reporting.
- Chemical proxies: Ammonia aligned well with operational PE_WWTP,TP_ (*R* ^2^ ∼ 0.99), but BOD and COD were biased for nonhuman inputs, particularly in agricultural catchments.
- Population concepts: Census-based headcounts and WWTP-reported residential PE capture contributing people, while load-based equivalents reflect operational burden. Misalignment between these concepts introduces interpretation challenges.
- Prescription data: NHS prescribing records exclude OTC and illicit use and assume uniform adherence, limiting the coverage of total pharmaceutical intake.
- Data infrastructure: National deployment requires harmonized shapefiles, prescribing records, and flow measurements, coordinated by agencies such as UKHSA or the Environment Agency.

#### 3.5.4 Future Perspectives

- National scaling: Integration with Environmental Agency catchments, 2021 Census, and NHS Digital data could underpin a harmonized UK-wide WBE population model.
- Dynamic adjustment: Incorporating real-time flow, commuting, and mobility data could provide rolling estimates for weekly or monthly WBE reporting.
- Improved proxies: Correction factors for land use and industrial discharges are needed to refine chemical-based PEs, especially COD and BOD.
- Demographic integration: Future frameworks should embed age structure, deprivation, and prescribing data into normalization, reducing the risk of bias in cross-site comparisons.
- Methodological innovation: Adaptive bootstrapping, Bayesian updating, or hybrid ML–Bayesian approaches may enhance scalability and robustness of fragmented catchments.

#### 3.5.5 Key Findings

- Method performance: The Density-Adjusted Bootstrap with Overlap (BtDO) provided the most precise and consistent estimates, particularly in large, high-overlap catchments.
- Population concept alignment: *P*_Census_ aligned with residential PE_WWTP,RP_ but consistently underestimated PE_WWTP,TP_.
- Chemical proxies: Ammonia tracked operational PEs closely (*R* ^2^ ∼ 0.99; ±6.6%), while COD and BOD were confounded by nonhuman sources.
- Demographic drivers: Age structure and deprivation were associated with higher pharmaceutical loads (e.g., analgesics and antidepressants), underscoring the importance of social context in WBE interpretation.
- Value of integration: Integration of census, NHS registrations, and prescription data improved demographic resolution and enabled more nuanced normalization of pharmaceutical loads.

## Supplementary Material



## Notes

Comprehensive details of the population estimation approaches used in this study, including the simple approach (SA), density-adjusted bootstrap (BtD), density-adjusted bootstrap with overlap (BtDO), and Bayesian hierarchical density (ByD) models; it includes methodological formulations, prior sensitivity analyses, model diagnostics, and statistical evaluations such as ANOVA and Tukey posthoc comparisons; supplementary tables present nomenclature, age and sex distributions, pharmaceutical compound lists, and correlation analyses linking demographic variables with pharmaceutical consumption; figures illustrate uncertainty metrics, model performance across wastewater catchments, land-use and water-quality relationships, and prescription trends by pharmaceutical family (PDF)
