# Supplementary material for: Demographic-Driven Wastewater-Based Epidemiology: Refining Population Estimates for Enhanced Chemical Monitoring
Source: Environ Sci Technol. 2025 Nov 18;59(47):25356–67. doi: 10.1021/acs.est.5c08178 (PMC12676738; doi:10.1021/acs.est.5c08178)
Supplement: Supplementary file 1 [file es5c08178_si_001.pdf]

Supporting Information

Demographic-Driven Wastewater-based Epidemiology: Refining Population Estimates for Enhanced Chemical Monitoring

Kishore Jagadeesan[1,2*], Megan Robertson[2,3], John Bagnall[2,3], and Barbara Kasprzyk-Hordern[1,2**]

[1]Department of Chemistry, University of Bath, Claverton Down, Bath, BA2 7AY, UK

[2]Centre of Excellence in Water-Based Early Warning Systems for Health Protection, Bath, BA2 7AY, UK

[3]Wessex Water, Bath, BA2 7WW, UK

Authors for correspondence:

* Kishore: kjj28@bath.ac.uk

** Barbara: bkh20@bath.ac.uk

Contents

S1. Terminology and Nomenclature (Methods 2.3) 4
S2. Population Estimation Approaches (Methods 2.4) 6
S4.1. Simple Approach (SA) [Methods 2.4.1] 6
S4.2. Density-Adjusted Approaches 6
S4.2.a. Density-Adjusted Bootstrap (BtD) [Methods 2.4.2.1] 6
S4.2.b. Density-Adjusted Bootstrap with Overlap (BtDO) [Methods 2.4.2] 6
S4.3. Bayesian Hierarchical Model (ByD) [Methods 2.4.3] 7
Rationale 7
Method 8
Priority and Fitting 8
Outputs 8
Outputs 8
S4.4. Comparative Performance Across Methods 9
S4.5. Bland–Altman Analysis with WWTP Equivalents 10
S4.6. Summary 10
S3. Uncertainty and Statistical Evaluation 11
S5.1. Uncertainty metrics 11
S5.2. Influence of Overlap Percentage 11
S5.3. ANOVA and Tukey Post-Hoc Comparisons 12
S5.4. Summary 13
S4. NHS GP Registration-Based Estimates [Methods 2.5] 13
S5. Mobility (Methods 2.6) 13
S6. Load-Based Population Equivalents (PE) [Methods 2.7] 15
S7. Demographic Profiles and Mobility (Methods 2.5) 16
S8. Land Use and Water Quality Correlations 17
S9. Pharmaceutical and Extended Correlation Analysis 18

Table S 1. Nomenclature 4
Table S 2: Age group distribution 16
Table S 3. Pharmaceuticals studied with abbreviations 19
Table S 4. Correlation analysis of pharmaceutical consumption across age groups. 21

Table of Figures

Figure S 1. BtDO interval width: BCa vs percentile 7
Figure S 2. ByD Prior sensitivity and Prior Predictive check 9
Figure S 3 Relative uncertainty across estimation methods by catchment. 9
Figure S 4. Bland–Altman: BtDO with $PE_{WWTP,TP}$ 10

Figure S5. Population Estimate Comparison 11
Figure S 6. Tukey HSD post-hoc comparisons of population estimation methods. 12
Figure S 7 Comparison of People Trave to work (%) patterns in WWTPs. 14
Figure S 8 Load-Based Population equivalents vs P_{Census} 15
Figure S 9. Land Usage Distribution by WWTP 17
Figure S 10. Correlation Plot: Land Usage % vs Water Quality Parameters 18
Figure S 11. WWTP catchment specific prescription rates of pharmaceuticals. 20

S1. Terminology and Nomenclature (Methods 2.3)

Table S 1. Nomenclature

Symbol / Acronym	Definition	Source / Notes
P	Population estimate (headcount-based)	Derived from census or NHS GP registration data
P_{Cenus}	Population estimate using Census data	Derived from census data
SA	Simple Approach Model	Census aggregation with overlap weighting
BtD	Bootstrap Density Model	Nonparametric bootstrap of census data
BtDO	Bootstrap Density with Overlap Adjustment Model	Extension of BtD accounting for catchment–census overlap
ByD	Bayesian Hierarchical Density Model	Posterior distributions incorporating density and overlap
P_{NHS}	Population estimates from NHS GP registrations	Captures registered patient population
PE_{WWTP}	Population equivalent (load-based)	Utility-reported annualised load-based population equivalent values.
$PE_{WWTP, RP}$	Population equivalent (load-based), Residential population	Utility-reported annualised load-based population equivalent values. (Residential)
$PE_{WWTP, TP}$	Population equivalent (load-based), Total operational equivalent	Utility-reported annualised load-based population equivalent values. (Total, includes trade effluent and tankered waste).
PE_{BOD}	Population equivalent based on biochemical oxygen demand	Utility operational data
PE_{COD}	Population equivalent based on chemical oxygen demand	Utility operational data
$PE_{Ammonia}$	Population equivalent based on ammonium loads	Utility operational data
OA	Output Area	Smallest UK census unit
LSOA	Lower Super Output Area	Aggregated UK census/health geography unit
CI	Confidence interval	95% intervals for bootstrap or Bayesian posterior estimates

Relative Uncertainty	Standardized measure of uncertainty (% of mean)	Used for method comparison
Margin of Error (MoE)	Half-width of CI relative to estimate	Expressed as %
LOO	Leave-one-out cross-validation	Model comparison metric
Posterior predictive check	Bayesian model calibration diagnostic	Based on simulated vs observed values
ANOVA	Analysis of variance	Statistical test for method comparison
Tukey HSD	Tukey’s Honestly Significant Difference	Post-hoc test for pairwise comparisons

S2. Population Estimation Approaches (Methods 2.4)

In this section we provide detailed formulations, validation checks, and supplementary figures supporting the population estimation approaches described in Methods 2.4. Figures S1-S4 present prior sensitivity, prior predictive checks, uncertainty analyses, and method comparisons to validate the robustness of the approaches across the four WWTP catchments.

S4.1. Simple Approach (SA) [Methods 2.4.1]

In the Simple Approach, let each output area (OA) i have population P_i and overlap percentage with a WWTP catchment $w_i \in [0,1]$. The total population estimate is PE_{SA} is calculated as follows,

$$PE_{SA} = \sum_{i=1}^{n} P_i w_i \qquad \text{Equation S1}$$

Where, $w_i = 1$ if OA is fully within the catchment, $w_i = 0$ if outside the catchment, $w_i \in [0,1]$ for partial overlap. Bounding totals were also derived (P_{min}, P_{max}) to assess allocation uncertainty.

As noted in the main text, SA showed the highest uncertainty, particularly in fragmented catchments. Relative uncertainties exceeded 20% in small catchments (e.g., WWTP C), compared with <3% in large, high-overlap catchments (e.g., WWTP A). Results are included in the comparative plots (Figures S4C and S4D).

S4.2. Density-Adjusted Approaches

S4.2.a. Density-Adjusted Bootstrap (BtD) [Methods 2.4.2.1]

The BtD method assumes that both overlap and density scale OA contributions. For OA i with population P_i and Area A_i, overlap fraction w_i and density $D_i = P_i/A_i$.

$$P_{BtD}^{(b)} = \sum_{i} P_i^{(b)} \cdot w_i \cdot D_i \qquad \text{Equation S2}$$

Bootstrap sampling (B = 1000) was used to quantify uncertainty. The final estimate was the bootstrap mean:

$$P_{BtD} = \frac{1}{B} \sum_{b=1}^{B} P_{BtD}^{(b)} ; B = 1000 \qquad \text{Equation S3}$$

Outputs included P_{BtD}, SD, CV, and 95% CIs. BtD reduced uncertainty compared to SA in large catchments but tended to overweight dense fully-contained OAs.

S4.2.b. Density-Adjusted Bootstrap with Overlap (BtDO) [Methods 2.4.2]

To address the limitation with BtD, BtDO scales only partially overlapping OAs by their density relative to catchment mean ($D_i/\overline{D}$), while fully overlapping OAs remain unscaled. For OA i

$$contrib_i = \begin{cases} P_i & w_i = 1 \\ P_i . w_i \cdot \dfrac{D_i}{\overline{D}} & 0 < w_i < 1 \end{cases} \qquad \text{Equation S4}$$

Catchment population in bootstrap iteration b:

$$P_{BtDO}^{(b)} = \sum_{i} contrib_i{}^{(b)} \qquad \text{Equation S5}$$

Final estimate:

$$P_{BtDO} = \frac{1}{B} \sum_{b=1}^{B} P_{BtDO}^{(b)} \qquad \text{Equation S6}$$

Uncertainty:

- SD across bootstrap replicates,
- CV = (SD / mean) × 100,
- 95% percentile CI (2.5th, 97.5th percentiles).

We used the boot package in R with 1000 iterations. In each replicate, the mean density $\overline{D}$ was recomputed from the intersecting OAs, ensuring density uncertainty was propagated. Outputs included P_{BtDO}, SD, CV, and 95% CIs. BtDO consistently produced narrower CIs and lower relative uncertainty than BtD. The advantage of BtDO is illustrated in Figure S1, where BCa confidence intervals were compared against percentile intervals. Results showed consistent performance with no evidence of bias from bootstrap interval choice.

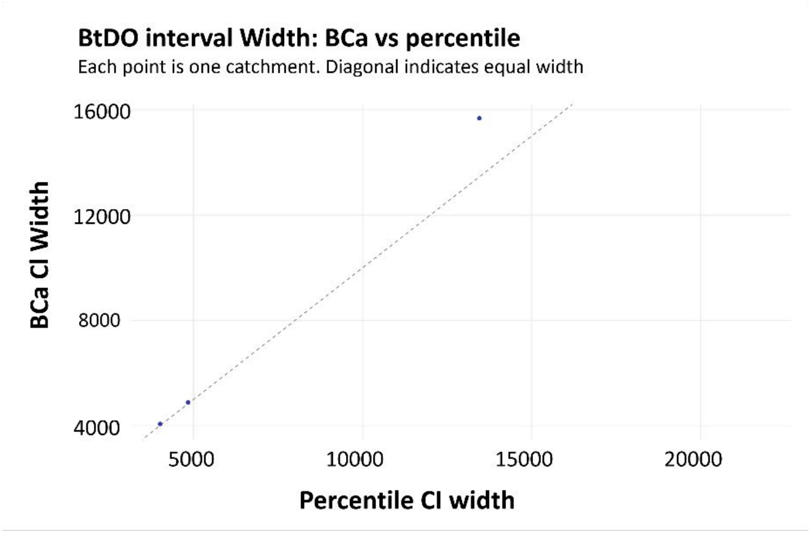

Figure S 1. BtDO interval width: BCa vs percentile
Comparison of interval widths for BtDO using percentile vs bias-corrected and accelerated (BCa) bootstrap. Each point represents a catchment; the 1:1 line indicates equal width. Most catchments fall close to the diagonal, confirming that percentile intervals are adequate.

S4.3. Bayesian Hierarchical Model (ByD) [Methods 2.4.3]

Rationale

Census overlay methods assume uniformity or a simple proportional role of density. In practice, density effects can be nonlinear and vary across contexts, such as, commuting patterns, multi-occupancy housing, and institutional populations. To capture this heterogeneity, we modelled density as strata-level random effects with partial pooling, alongside OA- and WWTP-level random effects. This framework

captures latent variation across density regimes while avoiding overfitting and provides a probabilistic treatment of uncertainty.

Method

Let i index OAs and j index WWTP catchments. Let k[i] denote the density stratum for OA i.

$$P_{ij} \sim Normal(\mu_{ij}, \sigma^2) \quad \text{Equation S7}$$

$$\mu_{ij} = \alpha + u_{k[i]}^{(density)} + u_i^{(OA)} + u_j^{(catchment)} \quad \text{Equation S8}$$

Random effects:

- $u_{k[i]}^{(density)} \sim Normal(0, \sigma_{density}^2)$, is the random effect for density stratum k (e.g. low, medium, high),
- $u_i^{(OA)} \sim Normal(0, \sigma_{OA}^2)$, random effect for OA i.
- $u_j^{(catchment)} \sim Normal(0, \sigma_{catchment}^2)$, random effect for WWTP catchment j,

Here P_{ij} is the contribution associated with OA i in catchment j. The linear predictor contains a global intercept α and exchangeable intercept shifts for density strata, OAs, and catchments.

Adjusted population density was binned into ordered strata (low to high), used as the grouping factor for $u_k^{(density)}$. This avoided fitting separate intercepts for every unique numeric density and improved both identifiability and interpretability.

Priority and Fitting

Weakly informative priors were used:$\alpha \sim Normal(0,100)$; random-effects SD~ $Normal(0,10)$; residual $\sigma \sim Normal(0,10)$. Models were fitted in R (brms) with 4 chains × 4000 iterations, setting acceptance probability (adapt_delta) to 0.95, to minimize divergent transitions. Convergence was confirmed by $\hat{R} < 1.01$, and effective sample sizes >1000. Posterior predictive checks indicated good model fit.

Outputs

Posterior samples were summarised as posterior means (PE_{ByD}), 95% credible intervals (CrIs), RMSE, and coverage probability for each catchment. Uncertainty was also expressed as RU(%), defined consistently across methods.

Outputs

- Prior sensitivity (Figure S2A): Posterior means changed by <5% when alternative SD priors were used, showing robustness to prior choice.
- Prior predictive checks (Figure S2B): Census totals lay within the simulated prior predictive distributions across all catchments, confirming that priors were weakly informative and not driving inference.

ByD produced wider intervals than BtDO but quantified uncertainty more comprehensively, particularly in small or fragmented catchments where census alignment was weak.

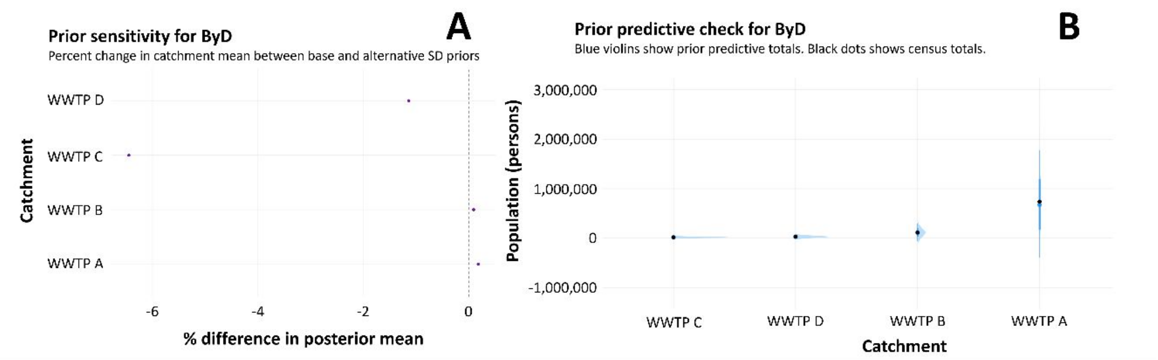

Figure S 2. ByD Prior sensitivity and Prior Predictive check
(S2A) Prior sensitivity for ByD, showing the percent change in posterior mean population estimates between base priors and alternative, more informative SD priors. Small differences (<5%) confirm robustness of the Bayesian model to prior specification. (S2B) Prior predictive check for ByD. Blue half-violin plots show prior predictive distributions for total population by catchment; black dots mark observed census totals. Census values fall within the prior predictive ranges, validating the weakly informative priors.

S4.4. Comparative Performance Across Methods

To evaluate relative performance across SA, BtD, BtDO, and ByD, we compared relative uncertainty and population estimates by catchment.

- Relative uncertainty (Figure S3): SA showed the highest uncertainty, BtDO the lowest, and ByD intermediate but stable across catchments.

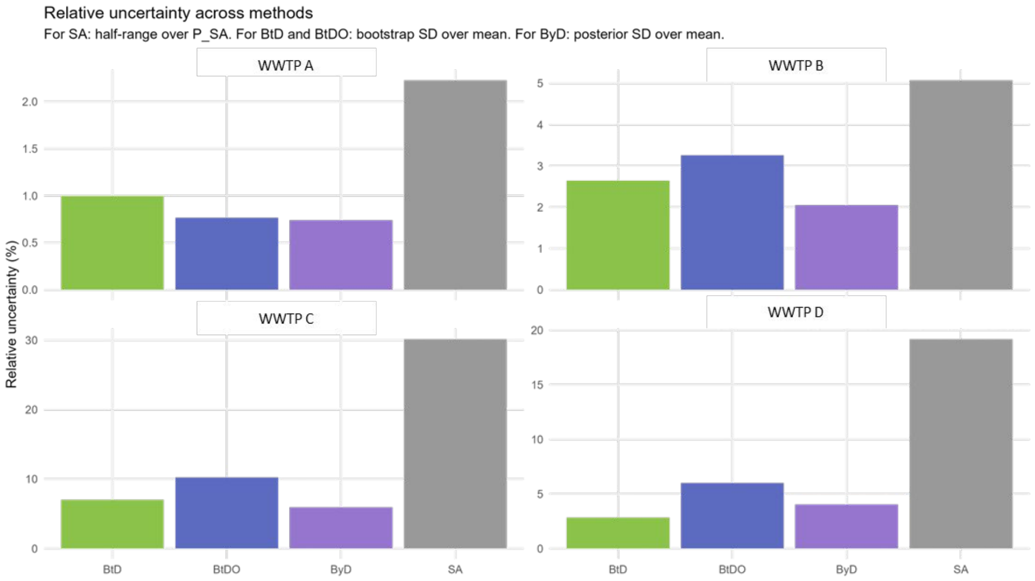

Figure S 3 Relative uncertainty across estimation methods by catchment. Uncertainty was defined as half-range over SA for the Simple Approach, bootstrap SD over mean for BtD and BtDO, and posterior SD over mean for ByD. SA shows substantially higher uncertainty, particularly in smaller or fragmented catchments, whereas BtDO and ByD yield narrower distributions.

S4.5. Bland–Altman Analysis with WWTP Equivalents

To further validate BtDO, we compared estimates against WWTP technical population equivalents ($PE_{WWTP,TP}$). The Bland–Altman analysis (Figure **S4**) showed small, unbiased differences across catchments, supporting BtDO as a practical balance between accuracy and feasibility.

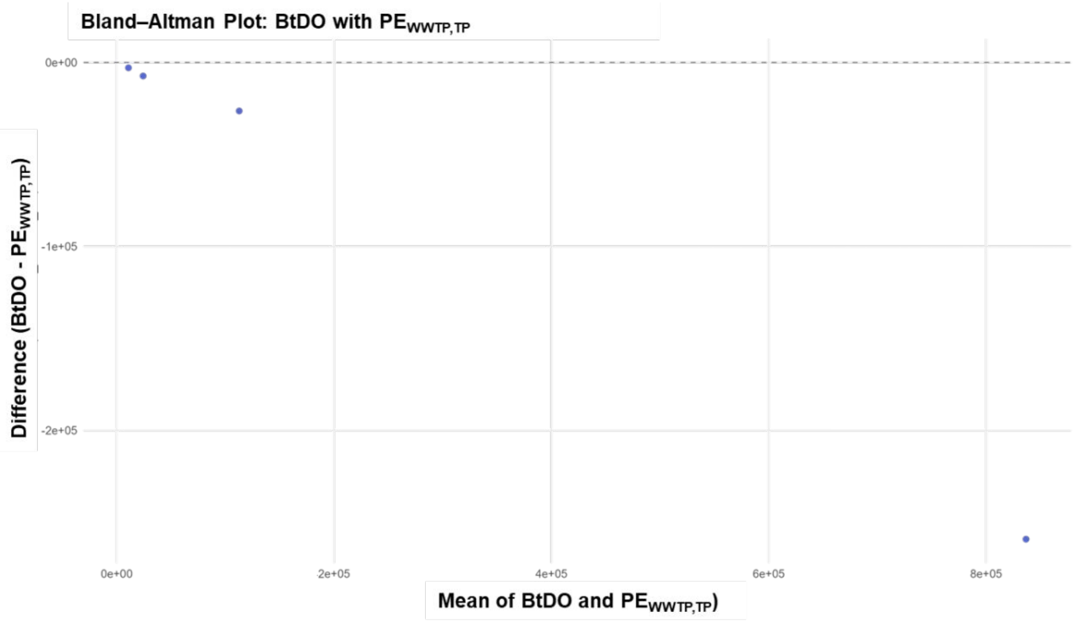

Figure S 4. Bland–Altman: BtDO with $PE_{WWTP,TP}$
Bland–Altman comparison of BtDO estimates with wastewater-based population equivalents ($PE_{WWTP,TP}$). Differences are small relative to method means, indicating consistency between demographic-based and water quality-based approaches.

S4.6. Summary

Supplementary Figures **S1–S4** confirm the validity of the methods:

- BtDO provided the most precise estimates in high-overlap catchments.
- ByD captured uncertainty comprehensively and was robust to prior specification.
- SA was sensitive to boundary misalignment and should be avoided in fragmented catchments.
- Comparative analyses showed that overlap percentage was a critical driver of uncertainty.

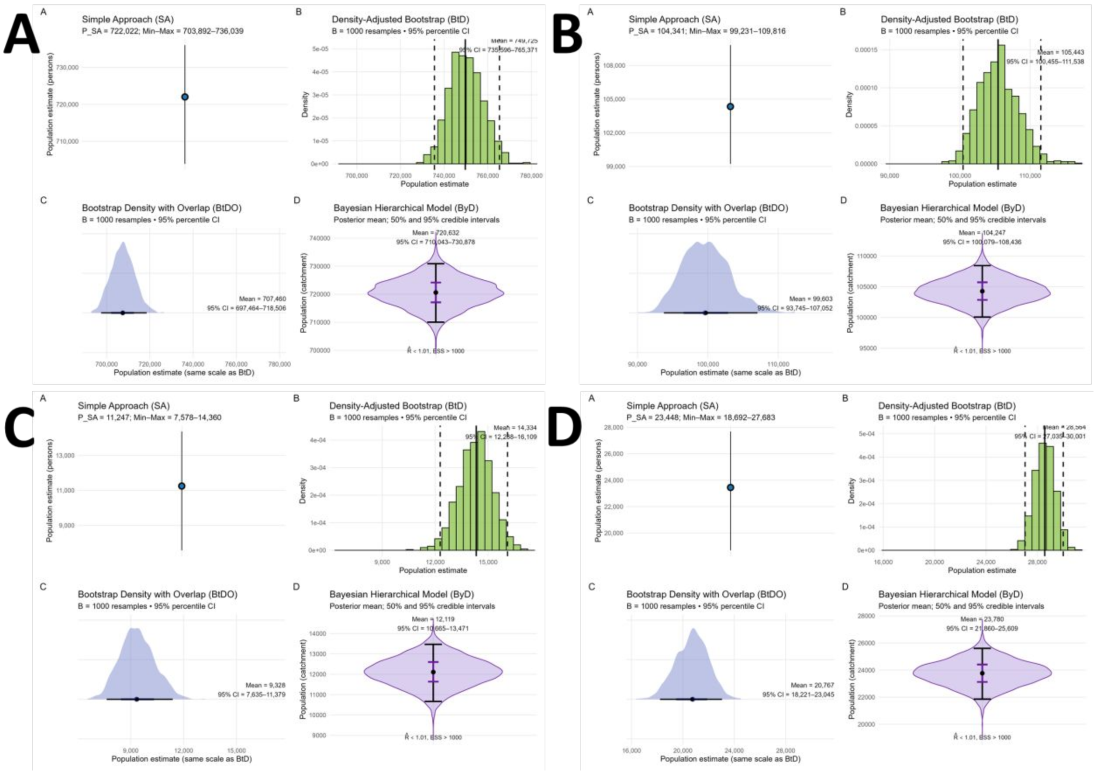

Figure S5. Population Estimate Comparison

Comparison of population estimation methods across four WWTP catchments (A-D). Each panel shows results from the Simple Approach (SA), Density-Adjusted Bootstrap (BtD), Density-Adjusted Bootstrap with Overlap (BtDO), and Bayesian Hierarchical Model (ByD). SA provides min–max ranges, BtD and BtDO show bootstrap distributions (B = 1000), and ByD presents posterior means with 95% credible intervals. BtDO yielded narrower uncertainty ranges in large, high-overlap catchments, while ByD captured broader uncertainty in smaller catchments with fragmented boundaries.

S3. Uncertainty and Statistical Evaluation

S5.1. Uncertainty metrics

We evaluated population estimation methods using three uncertainty metrics: absolute uncertainty, relative uncertainty, and margin of error. These were calculated consistently across methods as:

$$\text{Absolute Uncertainty} = \frac{\text{CI}_{\text{upper}} - \text{CI}_{\text{lower}}}{2} \qquad \text{Equation S10}$$

$$Relative\ Uncertainty\ (RU) = \frac{\text{Absolute Uncertainty}}{Estimate} \times 100 \qquad \text{Equation S11}$$

These measures provide a standardised basis for comparing method reliability across catchments of different sizes (Table 1).

S5.2. Influence of Overlap Percentage

Table 1 reports the number and percentage of Output Areas (OAs) with full overlap within each catchment. Higher overlap generally corresponded to lower relative uncertainty (e.g., WWTP A, 95% overlap, <2% RU; WWTP C, 42.6% overlap, >20%

RU). However, linear regression did not reveal a statistically significant correlation between RU and OA overlap percentage ($p > 0.05$). This non-significance likely reflects the limited sample size ($n = 4$ catchments), reducing statistical power.

S5.3. ANOVA and Tukey Post-Hoc Comparisons

A two-way ANOVA tested the effects of estimation method and OA overlap percentage on population estimates:

$$\text{Estimate} \sim \text{method} + \text{OA100Per}$$

Results showed significant differences between methods ($p = 0.0123$) and highly significant differences across overlap percentages ($p < 2e-16$), confirming that both methodological choice and spatial coverage influence estimate reliability.

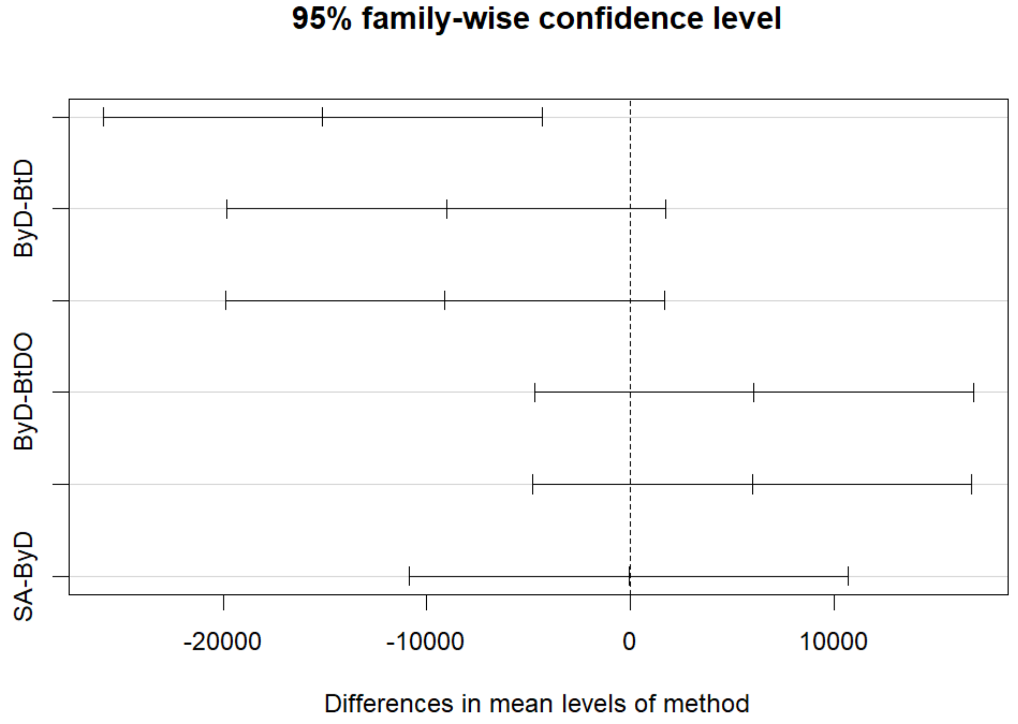

Figure S 6. Tukey HSD post-hoc comparisons of population estimation methods.
Horizontal lines show 95% family-wise confidence intervals for mean differences. Intervals not crossing zero indicate significant differences. ByD differed significantly from BtD and BtDO, while SA comparisons were not significant due to higher variance.

Tukey post-hoc tests provided pairwise comparisons (Figure S6). Results indicated:

- BtDO vs BtD: significant improvement when accounting for overlap ($p = 0.0079$), validating the refinement of scaling only partially overlapping OAs.
- Overlap levels: high-overlap catchments (≥90%) were significantly different from low-overlap catchments (≤50%) ($p < 0.0001$), reinforcing the importance of spatial alignment in reducing uncertainty.
- ByD vs SA: not statistically significant at the 0.05 level, reflecting comparable point estimates in some cases, though ByD provided richer uncertainty quantification.

- Other comparisons (e.g., ByD vs BtD) showed borderline significance, consistent with differences in uncertainty structures rather than mean estimates alone.

S5.4. Summary

Uncertainty was consistently lowest in BtDO (RU 1.5–20.1%), intermediate in BtD (4.2–33.2%), and highest in SA (2.4–36.6%). ByD produced moderate RU (12–18.5%) but offered richer probabilistic inference. Statistical tests confirm that overlap coverage is a critical determinant of estimate precision, and that BtDO significantly improves upon BtD by correcting density over-weighting.

S4. NHS GP Registration-Based Estimates [Methods 2.5]

GP registration counts (P_{NHS}) were compared against P_{Census}. Differences >10% were observed in catchments with high student or international worker populations. Limitations include double registrations and non-resident enrolees. NHS data were therefore treated descriptively, providing diagnostic insight into divergences rather than direct input to estimation models.

S5. Mobility (Methods 2.6)

Census 2021 travel-to-work data were used to calculate proportions commuting out, commuting in, and working from home by catchment. These metrics explained divergences between P_{Census} and P_{NHS}, particularly in catchments with high in-commuting. Stable populations in WWTPs A and B correspond with stronger census-NHS agreement. In contrast, WWTPs C and D showed higher proportions of commuters and international workers (>17%), consistent with overestimation by P_{NHS}. They were not included in the estimation models, but results are reported for context (Figures S7).

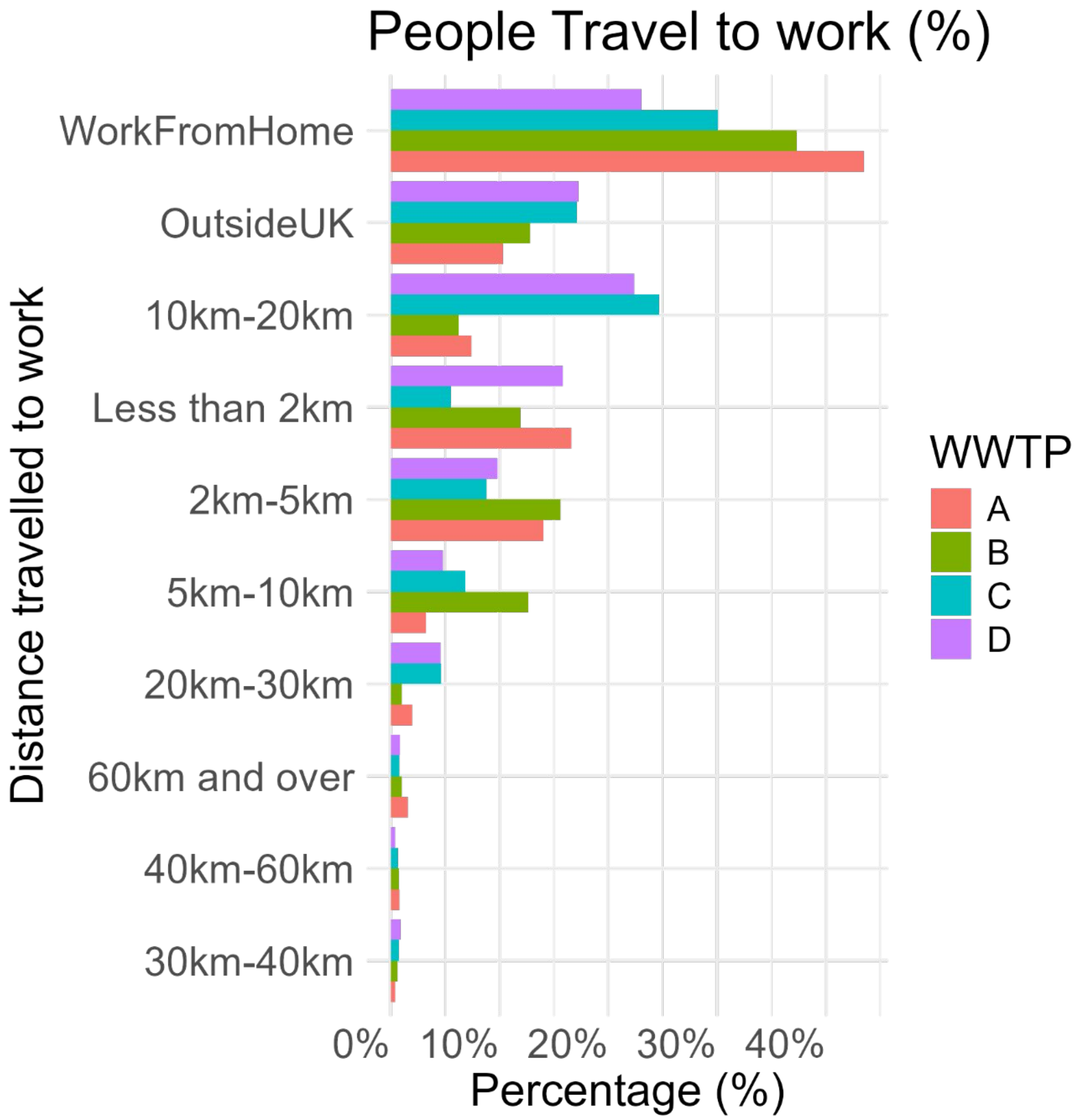

Figure S 7 Comparison of People Trave to work (%) patterns in WWTPs.
The bar plot illustrates the percentage of observed individuals grouped by travel distance categories for each WWTP.

S6. Load-Based Population Equivalents (PE) [Methods 2.7]

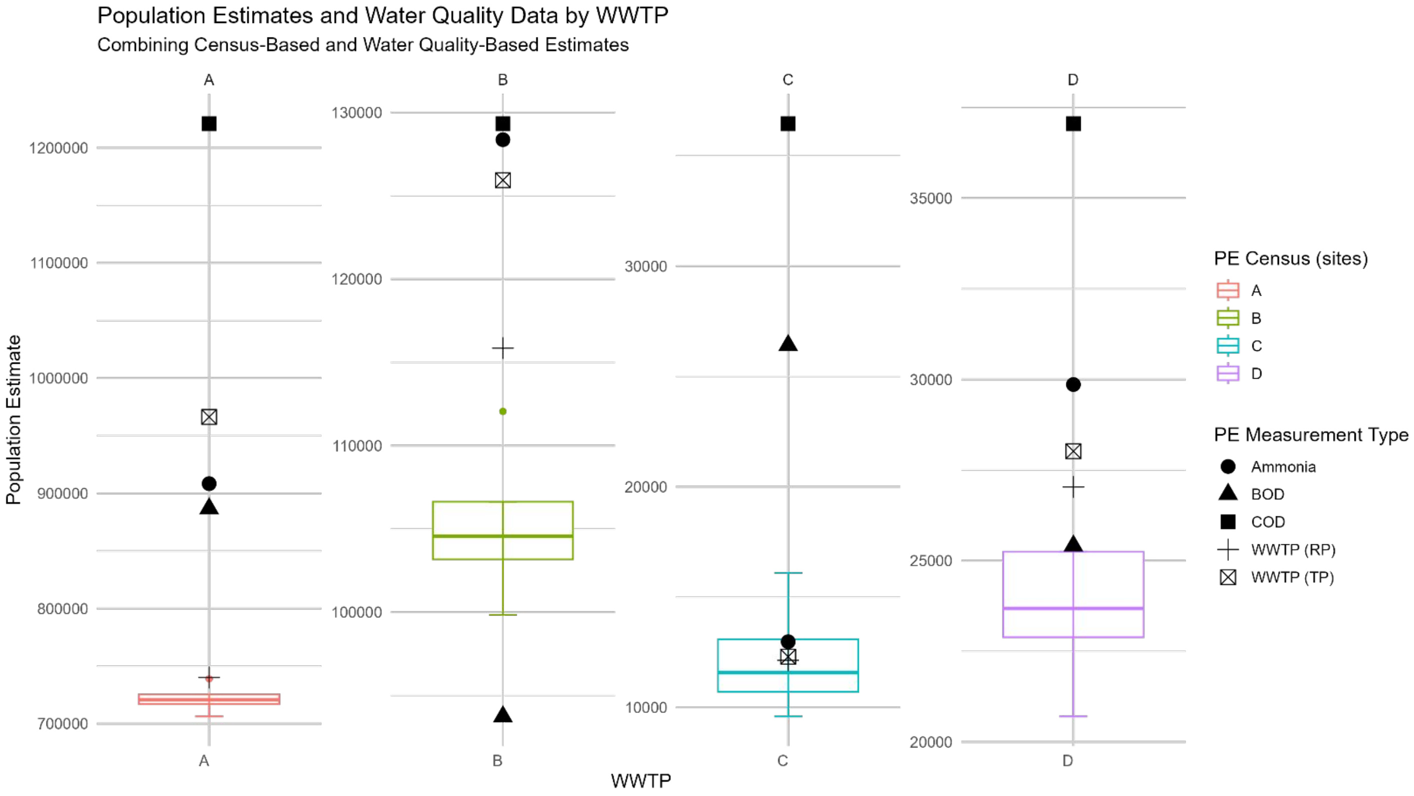

Figure S 8 Load-Based Population equivalents vs P_{Census}

Population estimates for four sites (A, B, C, and D) based on Ammonia (NH_4-N), Biological Oxygen Demand (BOD), Chemical Oxygen Demand (COD) measurements and Population equivalent from wastewater treatment plants representing resident population (WWTP(RP)) and total population (WWTP (TP)).

S7. Demographic Profiles and Mobility (Methods 2.5)

Table S 2: Age group distribution
Age Group by sex at each site, based on data from the 2021 Census.

WWTP	Sex	0-19 Years	20-49 Years	50-64 Years	65+ Years
A	Female	80932 ± 1909	166070 ± 3112	59431 ± 1886	56828 ± 2004
A	Male	84458 ± 1967	165454 ± 3050	59101 ± 1826	48324 ± 1755
B	Female	11841 ± 847	22421 ± 868	9338 ± 649	10111 ± 713
B	Male	12268 ± 751	21822 ± 782	8976 ± 616	8124 ± 636
C	Female	1260 ± 414	2018 ± 643	1109 ± 465	1218 ± 574
C	Male	1369 ± 422	1950 ± 620	1061 ± 430	1068 ± 482
D	Female	2644 ± 582	4280 ± 927	2421 ± 554	2645 ± 501
D	Male	2879 ± 616	4186 ± 888	2347 ± 515	2229 ± 436

S8. Land Use and Water Quality Correlations

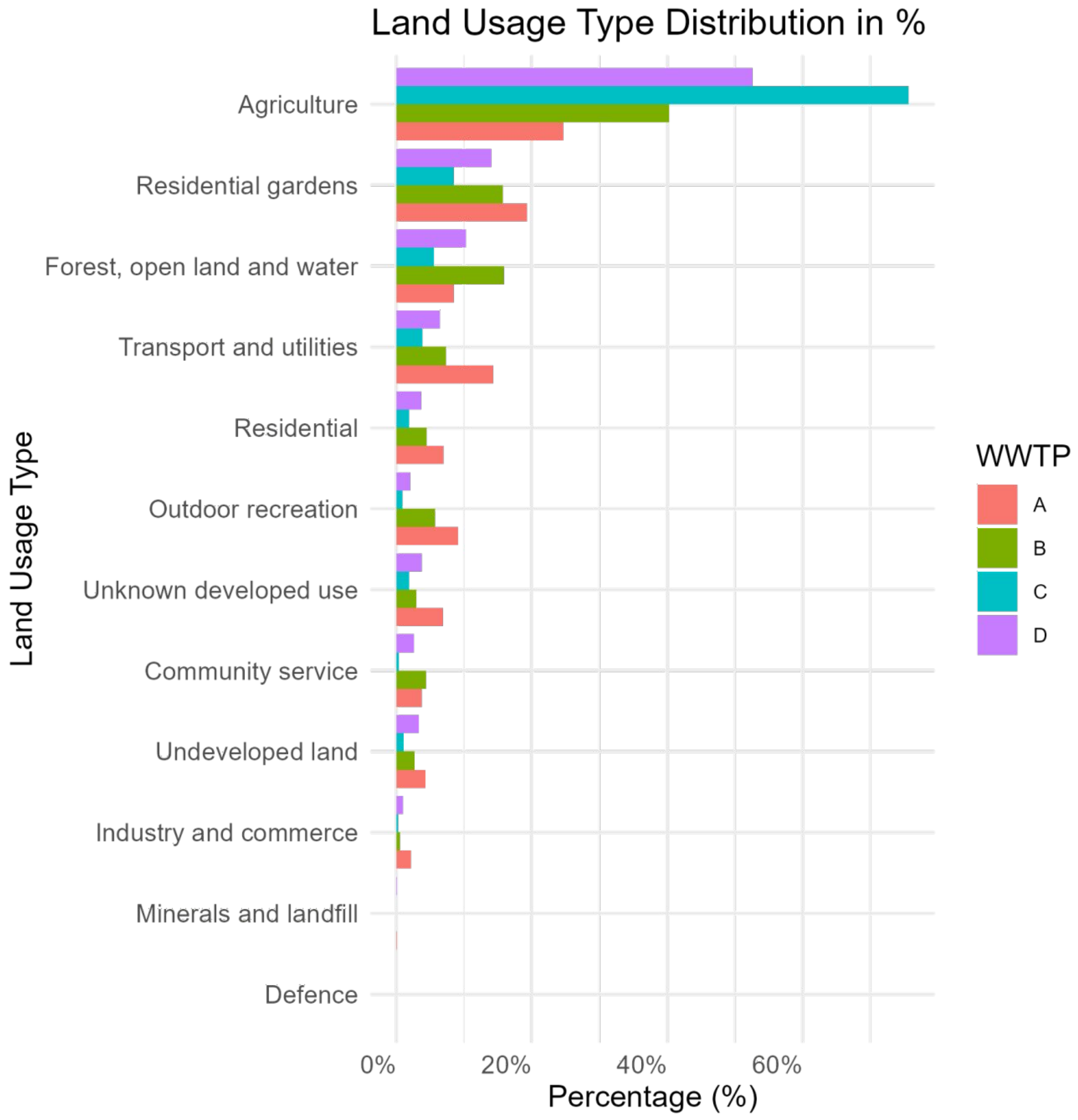

Figure S 9. Land Usage Distribution by WWTP

Land usage distribution by wastewater treatment plant (WWTP) catchment area. The bar plot shows the percentage of each land use type within each WWTP catchment, with land use types ordered based on the percentage.

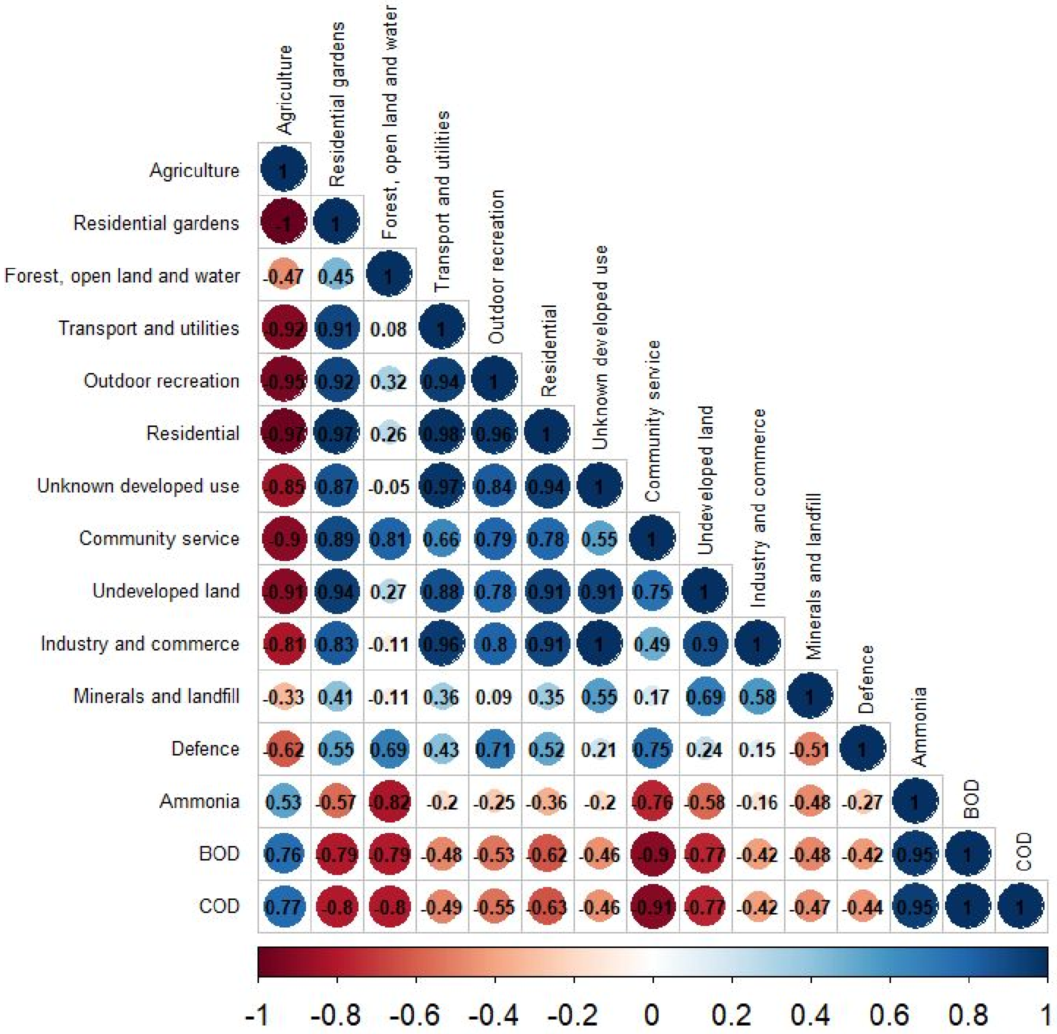

Figure S 10. Correlation Plot: Land Usage % vs Water Quality Parameters
Correlation plot showing the relationships between land use percentages and water quality parameters across wastewater treatment plant (WWTP) catchments. The Pearson correlation matrix is visualized as a lower triangular plot, with the strength and direction of correlations indicated by the size and colour of the circles. Positive correlations are shown in blue colours, while negative correlations are in red colours. The plot provides insight into the associations between different land use types and water quality parameters such as ammonia and BOD across the catchments

S9. Pharmaceutical and Extended Correlation Analysis

To investigate the relationship between average per capita pharmaceutical consumption and age demographics, a correlation analysis was conducted using data grouped by pharmaceutical family and age group (0–19, 20–49, 50–64, and 65+ years). The correlation coefficients were calculated using Pearson's or Spearman's methods, depending on the data distribution. Our approach utilized a custom R function, which dynamically selects between Pearson and Spearman

correlation methods based on data normality. This function performs Shapiro-Wilk tests for normality and chooses Pearson's correlation for normally distributed data and Spearman's for non-normal distributions. Statistical significance was determined using a two-tailed test, with a threshold of $p<0.05$. Groups with fewer than three valid observations were excluded from the analysis. Results were visualised in a heatmap, where correlation coefficients were represented using a diverging colour scale, and statistically significant correlations were marked with an asterisk (*).

Table S 3. Pharmaceuticals studied with abbreviations

S.No	Abbreviations	Pharmaceutical Compound	S.No	Abbreviations	Pharmaceutical Compound
1	ATL	Atenolol	22	KPF	Ketoprofen
2	ATR	Atorvastatin	23	LIS	Lisinopril
3	BEZ	Bezafibrate	24	MET	Metformin
4	BPL	Bisoprolol	25	MTP	Metoprolol
5	CBZ	Carbamazepine	26	MTZ	Metronidazole
6	CET	Cetirizine	27	NPX	Naproxen
7	CIM	Cimetidine	28	OFX	Ofloxacin
8	CTP	Citalopram	29	OXA	Oxazepam
9	CLR	Clarithromycin	30	ACE	Paracetamol
10	COD	Codeine	31	PRG	Pregabalin
11	DZP	Diazepam	32	PRO	Propranolol
12	DIC	Diclofenac	33	QTP	Quetiapine
13	DHC	Dihydrocodeine	34	RAN	Ranitidine
14	DIL	Diltiazem	35	SIL	Sildenafil
15	E2	Estradiol	36	SIT	Sitagliptin
16	EE2	Ethinylestradiol	37	SMX	Sulfamethoxazole
17	FEX	Fexofenadine	38	SLZ	Sulfasalazine
18	FXT	Fluoxetine	39	TEM	Temazepam
19	GPN	Gabapentin	40	TRA	Tramadol
20	GLI	Gliclazide	41	TMP	Trimethoprim
21	IBU	Ibuprofen	42	VAL	Valsartan
			43	VEN	Venlafaxine

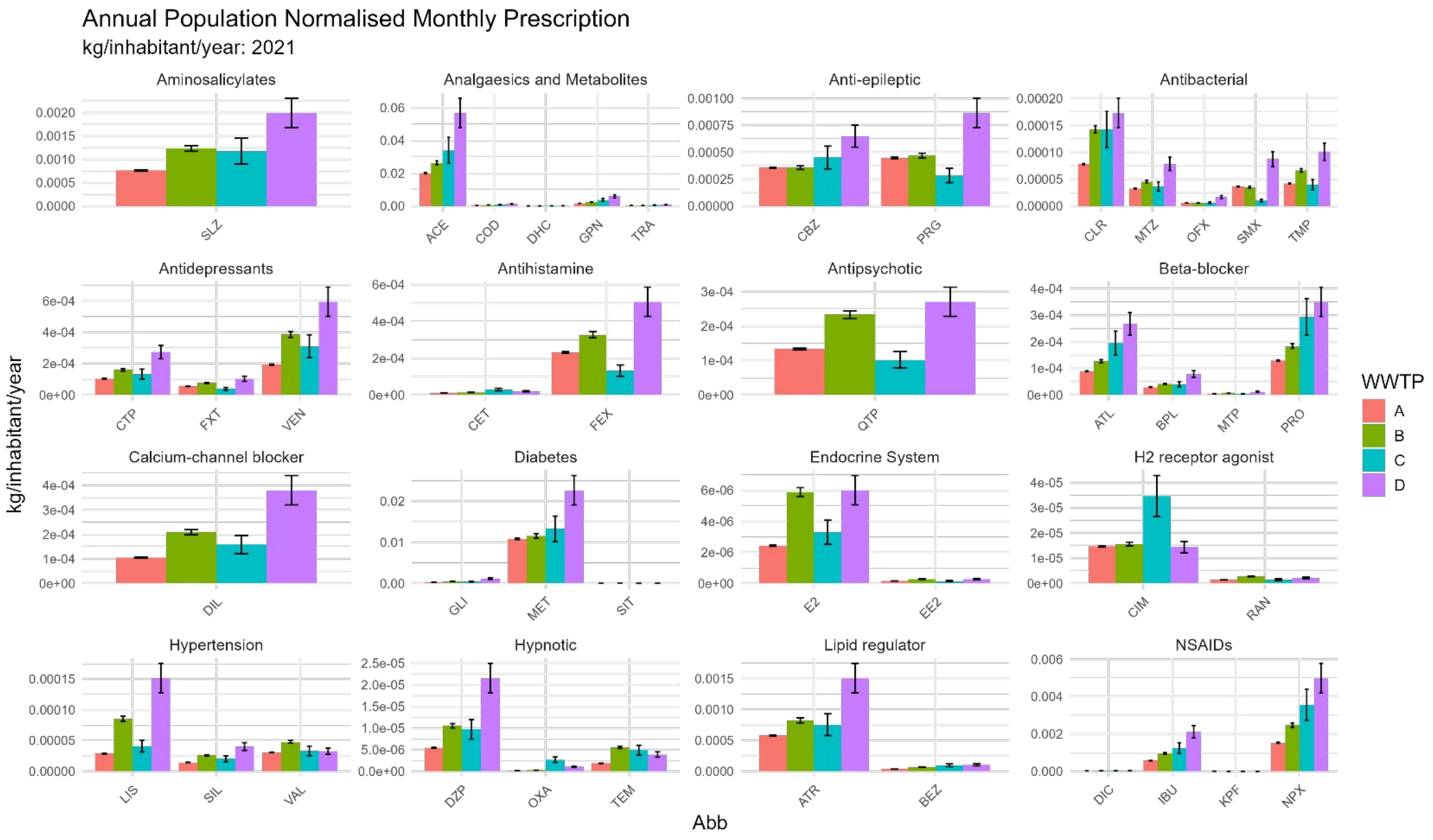

Figure S 11. WWTP catchment specific prescription rates of pharmaceuticals.

Bar plot displaying the population normalised prescription rates for various pharmaceutical families across different WWTPs in the year 2021. Prescription rates (kg/inhabitant/year) are shown on the y-axis, and individual pharmaceutical compound in x-axis. The data is grouped by pharmaceutical family, with each family displayed in separate facets.

Table S 4. Correlation analysis of pharmaceutical consumption across age groups.

Correlation coefficients (r) and their statistical significance ($p < 0.05$, indicated by *) for different pharmaceutical families across four age groups.

Pharmaceutical Family	Age Group	r	p-value	method	Significance
Amino salicylates	0-19 Years	0.19	0.66	Pearson	
Amino salicylates	20-49 Years	-0.79	0.02	Pearson	*
Amino salicylates	50-64 Years	0.83	0.01	Pearson	*
Amino salicylates	65+ Years	0.67	0.07	Pearson	
Analgesics and Metabolites	0-19 Years	0.27	0.52	Pearson	
Analgesics and Metabolites	20-49 Years	-0.89	0.00	Pearson	*
Analgesics and Metabolites	50-64 Years	0.92	0.00	Pearson	*
Analgesics and Metabolites	65+ Years	0.74	0.04	Pearson	*
Anti-Arrhythmic	0-19 Years	-	-	-	-
Anti-Arrhythmic	20-49 Years	-	-	-	-
Anti-Arrhythmic	50-64 Years	-	-	-	-
Anti-Arrhythmic	65+ Years	-	-	-	-
Anti-cancer	0-19 Years	0.29	0.49	Pearson	
Anti-cancer	20-49 Years	-0.93	0.00	Pearson	*
Anti-cancer	50-64 Years	0.95	0.00	Pearson	*
Anti-cancer	65+ Years	0.79	0.02	Pearson	*
Anti-depressant	0-19 Years	0.02	0.95	Spearman	
Anti-depressant	20-49 Years	-0.59	0.12	Spearman	
Anti-depressant	50-64 Years	0.69	0.06	Spearman	
Anti-depressant	65+ Years	0.44	0.28	Spearman	
Anti-epileptic	0-19 Years	0.02	0.95	Spearman	
Anti-epileptic	20-49 Years	-0.59	0.12	Spearman	
Anti-epileptic	50-64 Years	0.69	0.06	Spearman	

Anti-epileptic	65+ Years	0.44	0.28	Spearman	
Anti-hypertensive	0-19 Years	0.06	0.88	Pearson	
Anti- hypertensive	20-49 Years	-0.39	0.34	Pearson	
Anti- hypertensive	50-64 Years	0.49	0.22	Pearson	
Anti- hypertensive	65+ Years	0.28	0.49	Pearson	
Antibacterial	0-19 Years	0.15	0.73	Pearson	
Antibacterial	20-49 Years	-0.73	0.04	Pearson	*
Antibacterial	50-64 Years	0.77	0.02	Pearson	*
Antibacterial	65+ Years	0.62	0.10	Pearson	
Antidepressants	0-19 Years	0.02	0.95	Spearman	
Antidepressants	20-49 Years	-0.59	0.12	Spearman	
Antidepressants	50-64 Years	0.69	0.06	Spearman	
Antidepressants	65+ Years	0.44	0.28	Spearman	
Antifungal	0-19 Years	0.02	0.95	Spearman	
Antifungal	20-49 Years	-0.59	0.12	Spearman	
Antifungal	50-64 Years	0.69	0.06	Spearman	
Antifungal	65+ Years	0.44	0.28	Spearman	
Antihistamine	0-19 Years	-0.22	0.60	Spearman	
Antihistamine	20-49 Years	-0.10	0.82	Spearman	
Antihistamine	50-64 Years	0.25	0.56	Spearman	
Antihistamine	65+ Years	0.00	1.00	Spearman	
Antipsychotic	0-19 Years	0.02	0.95	Spearman	
Antipsychotic	20-49 Years	-0.59	0.12	Spearman	
Antipsychotic	50-64 Years	0.69	0.06	Spearman	
Antipsychotic	65+ Years	0.44	0.28	Spearman	
Antiviral Drugs	0-19 Years	-	-	-	-
Antiviral Drugs	20-49 Years	-	-	-	-

Antiviral Drugs	50-64 Years	-	-	-	-
Antiviral Drugs	65+ Years	-	-	-	-
Beta-blocker	0-19 Years	0.37	0.37	Spearman	
Beta-blocker	20-49 Years	-0.88	0.00	Spearman	*
Beta-blocker	50-64 Years	0.83	0.01	Spearman	*
Beta-blocker	65+ Years	0.78	0.02	Spearman	*
Bronchodilators	0-19 Years	0.20	0.64	Spearman	
Bronchodilators	20-49 Years	-0.88	0.00	Spearman	*
Bronchodilators	50-64 Years	0.93	0.00	Spearman	*
Bronchodilators	65+ Years	0.73	0.04	Spearman	*
CNS Stimulants and drugs used for ADHD	0-19 Years	-0.26	0.54	Spearman	
CNS Stimulants and drugs used for ADHD	20-49 Years	0.76	0.03	Spearman	*
CNS Stimulants and drugs used for ADHD	50-64 Years	-0.76	0.03	Spearman	*
CNS Stimulants and drugs used for ADHD	65+ Years	-0.63	0.09	Spearman	
Calcium-channel blocker	0-19 Years	0.14	0.74	Pearson	
Calcium-channel blocker	20-49 Years	-0.72	0.05	Pearson	*
Calcium-channel blocker	50-64 Years	0.77	0.03	Pearson	*
Calcium-channel blocker	65+ Years	0.61	0.11	Pearson	
Cough suppressant	0-19 Years	-0.26	0.54	Spearman	
Cough suppressant	20-49 Years	0.76	0.03	Spearman	*
Cough suppressant	50-64 Years	-0.76	0.03	Spearman	*
Cough suppressant	65+ Years	-0.63	0.09	Spearman	
Decongestants	0-19 Years	0.30	0.48	Spearman	
Decongestants	20-49 Years	-0.10	0.82	Spearman	
Decongestants	50-64 Years	-0.05	0.91	Spearman	
Decongestants	65+ Years	0.15	0.73	Spearman	
Dementia	0-19 Years	-0.21	0.62	Pearson	

Dementia	20-49 Years	0.11	0.79	Pearson	
Dementia	50-64 Years	-0.08	0.85	Pearson	
Dementia	65+ Years	-0.06	0.88	Pearson	
Diabetes	0-19 Years	0.20	0.64	Spearman	
Diabetes	20-49 Years	-0.88	0.00	Spearman	*
Diabetes	50-64 Years	0.93	0.00	Spearman	*
Diabetes	65+ Years	0.73	0.04	Spearman	*
Diuretics	0-19 Years	0.02	0.95	Spearman	
Diuretics	20-49 Years	-0.59	0.12	Spearman	
Diuretics	50-64 Years	0.69	0.06	Spearman	
Diuretics	65+ Years	0.44	0.28	Spearman	
Drugs Used in Nausea and Vertigo	0-19 Years	-0.26	0.54	Spearman	
Drugs Used in Nausea and Vertigo	20-49 Years	0.76	0.03	Spearman	*
Drugs Used in Nausea and Vertigo	50-64 Years	-0.76	0.03	Spearman	*
Drugs Used in Nausea and Vertigo	65+ Years	-0.63	0.09	Spearman	
Endocrine System	0-19 Years	-0.22	0.60	Spearman	
Endocrine System	20-49 Years	0.20	0.64	Spearman	
Endocrine System	50-64 Years	-0.10	0.82	Spearman	
Endocrine System	65+ Years	-0.24	0.56	Spearman	
H2 receptor agonist	0-19 Years	0.37	0.37	Spearman	
H2 receptor agonist	20-49 Years	-0.88	0.00	Spearman	*
H2 receptor agonist	50-64 Years	0.83	0.01	Spearman	*
H2 receptor agonist	65+ Years	0.78	0.02	Spearman	*
Hypertension	0-19 Years	0.07	0.87	Pearson	
Hypertension	20-49 Years	-0.58	0.13	Pearson	
Hypertension	50-64 Years	0.64	0.09	Pearson	
Hypertension	65+ Years	0.49	0.22	Pearson	

Hypnotic	0-19 Years	0.14	0.74	Pearson	
Hypnotic	20-49 Years	-0.72	0.04	Pearson	*
Hypnotic	50-64 Years	0.76	0.03	Pearson	*
Hypnotic	65+ Years	0.62	0.10	Pearson	
Lipid regulator	0-19 Years	0.20	0.64	Spearman	
Lipid regulator	20-49 Years	-0.88	0.00	Spearman	*
Lipid regulator	50-64 Years	0.93	0.00	Spearman	*
Lipid regulator	65+ Years	0.73	0.04	Spearman	*
NSAIDs	0-19 Years	0.34	0.41	Pearson	
NSAIDs	20-49 Years	-0.97	0.00	Pearson	*
NSAIDs	50-64 Years	0.98	0.00	Pearson	*
NSAIDs	65+ Years	0.82	0.01	Pearson	*
Nicotine Dependence	0-19 Years	-0.23	0.58	Pearson	
Nicotine Dependence	20-49 Years	0.14	0.74	Pearson	
Nicotine Dependence	50-64 Years	-0.08	0.84	Pearson	
Nicotine Dependence	65+ Years	-0.10	0.82	Pearson	
Other Antianginal Drugs	0-19 Years	-0.22	0.60	Spearman	
Other Antianginal Drugs	20-49 Years	-0.10	0.82	Spearman	
Other Antianginal Drugs	50-64 Years	0.25	0.56	Spearman	
Other Antianginal Drugs	65+ Years	0.00	1.00	Spearman	
Obesity	0-19 Years	0.20	0.64	Spearman	
Obesity	20-49 Years	-0.88	0.00	Spearman	*
Obesity	50-64 Years	0.93	0.00	Spearman	*
Obesity	65+ Years	0.73	0.04	Spearman	*

1. Tscharke, B. J.; O'Brien, J. W.; Ort, C.; Grant, S.; Gerber, C.; Bade, R.; Thai, P. K.; Thomas, K. V.; Mueller, J. F., Harnessing the Power of the Census: Characterizing Wastewater Treatment Plant Catchment Populations for Wastewater-Based Epidemiology. *Environ. Sci. Technol.* **2019,** *53* (17), 10303-10311.
2. Price, M.; Tscharke, B.; Chappell, A.; Kah, M.; Sila-Nowicka, K.; Morris, H.; Ward, D.; Trowsdale, S., Testing methods to estimate population size for wastewater treatment plants using census data: Implications for wastewater-based epidemiology. *Sci Total Environ* **2024,** *922*, 170974.
